# Integrative Multi-Omics and Single-Cell Analyses Identify *FKBP10* as a CAF-Associated Biomarker Linked to ECM Remodeling and Aggressiveness in Neuroblastoma

**DOI:** 10.32604/or.2026.087891

**Published:** 2026-10-28

**Authors:** Zhaolong Wang, Baocheng Gong, Chaoyu Wang, Zhongyuan Li, Shuai Man, Na Lei, Jiarun Fu, Yan Jin, Benfu Zhong, Qiang Zhao

**Affiliations:** Department of Pediatric Oncology, Tianjin Medical University Cancer Institute & Hospital, National Clinical Research Center for Cancer, Tianjin’s Clinical Research Center for Cancer, Key Laboratory of Cancer Prevention and Therapy, Tianjin, China

**Keywords:** Neuroblastoma, *FKBP10*, cancer-associated fibroblasts, tumor microenvironment, extracellular matrix, tumor progression

## Abstract

**Objectives:** Neuroblastoma (NB) is a heterogeneous pediatric malignancy driven by both intrinsic oncogenic programs and the stromal microenvironment. However, the critical stromal regulators associated with poor prognosis and metastasis in NB remain poorly characterized. This study aimed to identify key cancer-associated fibroblast (CAF)-related genes and to investigate the clinical significance, biological function, and molecular mechanism of *FKBP10* in NB malignant progression. **Methods:** Multi-cohort bioinformatic analyses, including weighted gene co-expression network analysis, machine learning-based *LASSO-Cox* modeling, and single-cell transcriptomics were performed to screen core stromal biomarkers. *In vitro* functional assays, rescue experiments, drug sensitivity assays, and *in vivo* xenograft models were used to validate the oncogenic role of *FKBP10* in NB. **Results:** High CAF infiltration correlated with unfavorable prognosis in NB. *FKBP10* was identified as a core stromal effector highly expressed in fibroblasts and malignant NB cells. The *FKBP10*-based signature effectively stratified patient prognosis in primary and validation cohorts. Mechanistically, *FKBP10* facilitated extracellular matrix (ECM) remodeling and mesenchymal reprogramming, accompanied by tumor immune evasion and therapeutic resistance. Functional assays demonstrated that *FKBP10* depletion suppressed NB proliferation, migration, invasion, and tumor growth, while restoring *FKBP10* rescued malignant phenotypes and reduced chemosensitivity. **Conclusions:** *FKBP10* promotes aggressive NB progression by regulating stromal activation, ECM remodeling and malignant behaviors. *FKBP10* serves as a reliable prognostic biomarker and a promising therapeutic target for high-risk neuroblastoma.

## 1 Introduction

Neuroblastoma (NB) is the most common extracranial solid tumor of childhood and remains one of the leading causes of pediatric cancer-related mortality [1,2,3,4]. Originating from neural crest-derived sympathoadrenal precursors, NB exhibits marked clinical and biological heterogeneity, ranging from spontaneous regression to highly aggressive metastatic disease [1,5,6,7]. Despite advances in multimodal therapy, including intensive chemotherapy, surgery, radiotherapy, autologous stem-cell transplantation, differentiation therapy, and anti-GD2 immunotherapy, the prognosis of patients with high-risk NB remains unsatisfactory, particularly in those with metastatic relapse or refractory disease [2,3,4,8,9]. These persistent clinical challenges highlight the need to better understand the biological processes associated with NB progression and to identify biomarkers that may improve risk stratification and therapeutic development [3,5,8].

Accumulating evidence indicates that the tumor microenvironment (TME), including both stromal and immune components, is closely associated with tumor progression, metastatic behavior, and therapeutic response [10,11,12,13]. Among stromal populations, cancer-associated fibroblasts are increasingly recognized as key regulators of extracellular matrix remodeling, tumor cell invasion, metastatic dissemination, and treatment resistance [14,15,16,17,18]. In addition, increasing recognition of cancer-associated fibroblast (CAF) heterogeneity suggests that distinct fibroblast states may differentially influence matrix organization, inflammatory signaling, immune regulation, and treatment response [17,19,20,21,22]. These observations raise the possibility that fibroblast-associated transcriptional programs may provide insight into the biological features of aggressive NB [10,12,13].

Extracellular matrix (ECM) remodeling represents an important aspect of tumor progression and has been linked to invasive phenotypes in multiple malignancies. Transcriptional programs involving focal adhesion, *TGF-β* signaling, and mesenchymal transition-like states are frequently observed in tumors with enhanced migratory and invasive properties [19,23,24,25,26,27]. The ECM is no longer viewed as a passive structural scaffold but rather as a dynamic and instructive component of the TME [23,24,28]. Matrix remodeling, collagen deposition, and altered stromal organization can actively regulate tumor cell migration, metastatic dissemination, and adaptation to microenvironmental stress [24,26,28,29]. Because CAFs are major producers and remodelers of ECM, stromal factors involved in matrix organization may be particularly relevant to understanding the progression of NB [14,16,18,25].

*FKBP10*, an endoplasmic reticulum-resident chaperone of the *FK506*-binding protein family, participates in procollagen folding and matrix assembly [30,31]. Emerging evidence in human malignancies has implicated *FKBP10* in tumor progression and adverse clinical behavior [32,33,34,35]. Given its close functional relationship with collagen maturation and stromal remodeling, *FKBP10* is biologically well positioned to link CAF-associated matrix remodeling with tumor aggressiveness [30,31,32]. However, the relevance of *FKBP10* to NB remains poorly defined. In particular, it is unclear whether *FKBP10* expression is associated with CAF-enriched microenvironments, ECM-remodeling programs, and adverse clinical outcomes in NB. Clarifying these issues may improve our understanding of stromal-associated features in aggressive NB and help identify candidate biomarkers for high-risk disease.

In this study, we aimed to identify key CAF- and stroma-related genes associated with neuroblastoma progression and poor prognosis, and to determine their biological and clinical significance. To achieve this, we integrated multi-cohort bulk transcriptome data, single-cell sequencing analysis, weighted gene co-expression network analysis, and machine learning algorithms to screen candidate stromal functional genes. We then focused on *FKBP10* and sought to clarify its prognostic value, its role in regulating malignant phenotypes and therapeutic sensitivity in neuroblastoma, and the underlying molecular mechanisms through *in vitro* experiments and *in vivo* xenograft models.

## 2 Materials and Methods

### 2.1 Estimation of CAF Infiltration and Survival Analysis

Transcriptomic profiles and corresponding clinical survival data of neuroblastoma patients were retrieved from the GSE49710 cohort in the Gene Expression Omnibus (GEO) database and the E-MTAB cohort in the ArrayExpress database. The GSE49710 cohort comprised 498 primary neuroblastoma samples profiled using the Agilent-020382 Human Custom Microarray 44K platform (GPL16876), with available clinical variables including age at diagnosis, sex, MYCN amplification status, INSS stage, high-risk status, disease progression, survival status, and follow-up information. The E-MTAB cohort included 223 neuroblastoma samples profiled using the Agilent-020382 Human Custom Microarray 44K platform, with available information on age at diagnosis, MYCN amplification status, INSS stage, overall survival status, and survival time. CAF enrichment scores were calculated separately in the GSE49710 and E-MTAB cohorts using the xCell R package (v1.1.0), with the built-in reference signatures and default parameters. Results from the two cohorts were analyzed independently and compared for consistency. Subsequently, patients in both cohorts were dichotomized into high- and low-CAF infiltration groups according to the median CAF enrichment score. Kaplan-Meier curves were generated to evaluate overall survival (OS), and differences between groups were compared using the log-rank test. Hazard ratio (HR) and 95% confidence interval (CI) were estimated using univariate Cox proportional hazards regression to assess the prognostic risk associated with CAF enrichment.

### 2.2 Weighted Gene Co-Expression Network Analysis (WGCNA)

Weighted Gene Co-expression Network Analysis (WGCNA) was applied to the transcriptomic profiles of four independent neuroblastoma cohorts (TARGET, GSE45547, GSE49710, and E-MTAB). After normalization and removal of low-variance genes, signed co-expression networks were constructed using cohort-specific soft-thresholding powers selected according to the scale-free topology criterion. Modules were identified with a minimum module size of 30 genes and merged at a cut height of 0.25. The four datasets were analyzed independently and were not combined; therefore, cross-cohort batch correction was not performed. After determining the optimal soft-thresholding powers to construct scale-free topological networks, module-trait relationship analyses were performed. The phenotypic traits, specifically stromal and CAF infiltration scores, were quantified using multiple computational algorithms, including EPIC (v1.1.7), MCPcounter (v1.2.0), and xCell (v1.1.0). Gene identifiers were converted to HUGO gene symbols, and the built-in reference signatures and default parameters were used. Results from the three methods were analyzed separately and compared for consistency. Pearson correlations were calculated between module eigengenes and stromal metrics, and *p*-values were adjusted for multiple testing using the Benjamini–Hochberg method, with an FDR < 0.05 considered significant. Ultimately, CAF-associated modules were defined as those showing positive correlations with CAF-related scores across all three algorithms, with Pearson r > 0.20 and Benjamini–Hochberg-adjusted FDR < 0.05. When multiple modules met these criteria, the module with the highest mean correlation coefficient across the three methods was selected for downstream analysis.

### 2.3 Feature Selection via a Multi-Algorithm Machine Learning Framework

A consensus set of candidate genes was initially established by intersecting the highly correlated CAF module genes derived from the four independent WGCNA cohorts. To further prioritize the core features, Candidate genes derived from CAF-associated modules were then used as predictors to classify patients into high- and low-CAF groups. GLM, RF, SVM, and XGB models were developed. Continuous predictors were standardized before modeling. 10-fold cross-validation was used for model tuning, with a random seed of 123. Hyperparameters were optimized by cross-validation, and model performance was evaluated in the held-out test set. The models were developed in the GSE49710 cohort and independently validated in the E-MTAB cohort, with performance assessed by residual distribution analysis (quantified by the root mean square error [RMSE]), and receiver operating characteristic (ROC) curves (assessed by the area under the curve [AUC]). In addition, the relative importance of individual features in each respective model was ranked according to the increase in RMSE following feature permutation. The SVM model showed the highest AUC (0.938), followed by RF (0.915), XGB (0.891), and GLM (0.786), and was therefore selected as the optimal model. The highest-ranked SVM features were retained as core CAF-associated genes for subsequent survival analysis.

### 2.4 Construction of the LASSO Prognostic Risk Model

To evaluate the prognostic implications of the top candidate features selected by the SVM algorithm, the prognostic model was constructed in the GSE49710 cohort (*n* = 498) and validated in the E-MTAB cohort (*n* = 223). Patients with available gene expression and overall survival data were included. Cox regression was used to estimate HR and 95% CI for the SVM-selected genes. Subsequently, to eliminate redundant features and construct an optimal prognostic risk model, the prognostically significant genes (FDR < 0.05, Benjamini–Hochberg correction) were incorporated into a Least Absolute Shrinkage and Selection Operator (*LASSO*) Cox regression analysis. *LASSO Cox* regression was performed using the glmnet R package (v4.4.1) with the cv.glmnet function (family = “cox”, alpha = 1). The optimal λ was selected by 10-fold cross-validation using the minimum partial likelihood deviance criterion (lambda.min). Ultimately, at lambda.min, FKBP10 and MFAP4 retained non-zero coefficients and were used to construct the prognostic signature. The risk score was calculated as follows: Risk score = 0.4913 × FKBP10 expression + 0.2501 × MFAP4 expression.

### 2.5 Validation of the Risk Signature and Tumor Microenvironment Analysis

To evaluate the prognostic performance of the established risk signature, the prognostic signature was developed in the GSE49710 cohort and validated in the independent E-MTAB cohort. Patients in both cohorts were stratified into high- and low-risk groups using the cutoff derived from GSE49710. Kaplan-Meier survival curves were generated to compare overall survival between the two risk groups, and statistical significance was assessed using the log-rank test. Univariate and multivariate Cox proportional hazards regression analyses were performed to assess the prognostic value of the risk score. The multivariate model included age, MYCN status, and INSS stage as covariates. HR and 95% CI were calculated. The predictive accuracy of the risk signature was further evaluated using time-dependent receiver operating characteristic curves and the concordance index.

To explore the biological relevance of the risk signature to the tumor microenvironment, correlations between risk scores and TME-related features, including stromal components, immune cell infiltration, fibroblast/CAF-related signatures, and immune evasion scores, were systematically analyzed. These features were inferred using EPIC (v1.1.7), MCPcounter (v1.2.0), xCell (v1.1.0), ESTIMATE (v1.0.13), and the Tumor Immune Dysfunction and Exclusion (TIDE) web server with default settings. Spearman’s rank correlation analysis was used to evaluate associations between the risk score and TME features. *p*-values from all TME-related correlation analyses were adjusted using the Benjamini–Hochberg method, with an FDR < 0.05 considered significant.

### 2.6 Prediction of Immunotherapeutic Response Utilizing the TIDE Algorithm

To evaluate the propensity for immune evasion and predict the clinical response to immune checkpoint blockade (ICB), the Tumor Immune Dysfunction and Exclusion (TIDE) algorithm was employed. Because TIDE has not been specifically validated for predicting ICB response in neuroblastoma, these results were interpreted as supportive rather than definitive evidence of immunotherapy responsiveness. Based on the established prognostic model, patients were stratified into high- and low-risk groups. Continuous TIDE scores and the predicted dichotomous outcomes (patients with TIDE scores < 0 were classified as predicted responders, whereas those with scores > 0 were classified as non-responders) were derived for each patient and systematically compared between the two risk strata. Furthermore, to quantitatively assess the discriminative accuracy of the risk signature in predicting immunotherapeutic efficacy, ROC analysis was used to evaluate the ability of the risk score to distinguish TIDE-predicted responders from non-responders. The endpoint was the TIDE-derived response category rather than clinically observed immunotherapy response. AUCs and 95% CI were calculated using the pROC R package, with confidence intervals estimated by the DeLong method.

### 2.7 Prediction of Therapeutic Sensitivity to Pharmacological Agents

To evaluate the potential of the established risk signature in predicting pharmacological efficacy, patients were initially stratified into high- and low-risk groups. Drug sensitivity was computationally predicted using the oncoPredict R package (v1.3.1) with GDSC2 cell-line expression and drug-response data as the reference. Normalized gene expression matrices were used as input to the calcPhenotype function, with ComBat batch correction, removal of the lowest 20% least variable genes, and all other parameters set to defaults. The resulting values represent predicted drug sensitivity rather than experimentally measured responses. Specifically, the predicted sensitivity scores for broad-spectrum chemotherapeutic drugs (including cisplatin, 5-fluorouracil, topotecan, and irinotecan) and specific targeted inhibitors (lapatinib and MK-2206) were systematically derived. Subsequently, to characterize the drug-resistant phenotype, these continuous sensitivity scores were statistically compared between the high- and low-risk groups using the Wilcoxon rank-sum test. *p*-values across the tested drugs were adjusted using the Benjamini–Hochberg method, with an FDR < 0.05 considered significant.

### 2.8 Single-Cell RNA Sequencing Analysis and AUCell Enrichment Scoring

Single-cell RNA-seq data were obtained from GSE287352, comprising 20 neuroblastoma-related tumors profiled using the Singleron GEXSCOPE platform. Cells with 200–5000 detected genes, fewer than 30,000 UMIs, and mitochondrial transcript proportions ≤ 30% were retained. No additional nFeature_RNA/nCount_RNA ratio cutoff was applied. After quality control, 89,882 cells were retained for downstream analysis. Single-cell data were processed using Seurat (v5.5.0). Each sample was normalized using LogNormalize with a scale factor of 10,000, and the top 2000 highly variable genes were selected using the vst method. Batch effects among samples were corrected by Seurat anchor-based integration using FindIntegrationAnchors and IntegrateData with the first 30 dimensions. The integrated data were scaled and subjected to PCA, followed by UMAP visualization using the first 30 principal components. PCA was performed on the highly variable genes, and the first 30 principal components were used for nearest-neighbor graph construction and UMAP visualization. Cell clusters were identified using the Louvain algorithm with a resolution of 0.5. UMAP was run with 30 neighbors and a minimum distance of 0.3. Cell types were annotated according to canonical markers, including PHOX2B, TH, DBH, CHGA, and HAND2 for neuroblastoma cells; CD3D/CD3E for T cells; MS4A1/CD79A for B cells; LST1/TYROBP for myeloid cells; C1QA/C1QB for macrophages; PECAM1/VWF for endothelial cells; COL1A1/COL1A2/DCN for fibroblasts; and SOX10/S100B/PLP1 for Schwann cells. Finally, to determine the exact cellular origin and spatial distribution of the established risk signature within the complex TME, AUCell analysis was performed using the AUCell R package (v1.34.0) to assess the enrichment of cell-type marker gene sets in individual cells. Marker sets included T cells (CD3D, CD3E, TRAC, CD2), epithelial cells (EPCAM, KRT8, KRT18, CDH1), natural killer (NK) cells (NCAM1, GNLY, NKG7, GZMB), monocytes (CD14, LYZ, S100A8, S100A9), macrophages (CD68, AIF1, CSF1R, CD163), endothelial cells (PECAM1, VWF, CDH5, ENG, CLDN5), smooth muscle cells (ACTA2, MYH11, TAGLN, CNN1), B cells (MS4A1, CD19, CD79A, PAX5), neuroblastoma cells (PHOX2B, TH, DBH, SYP, CHGA, MYCN, TOP2A), and dendritic cells (IL3RA, CLEC4C, NRP1, GAD2). AUC scores were calculated using the default settings and visualized via a hierarchical clustering heatmap.

### 2.9 Cellular Localization and Signal Amplification Analysis of FKBP10

To investigate the *in situ* expression pattern and cellular localization of the core hub gene *FKBP10*, its transcript distribution was projected onto the established UMAP manifold using feature plot visualization, with expression levels represented by log-normalized values. Furthermore, to precisely delineate and quantify the specific cellular origins of *FKBP10* within the complex TME, *FKBP10*-expressing cells were identified using a normalized expression threshold of >0.25. Cell populations were considered *FKBP10*-enriched only when *FKBP10* was detected in at least 10% of cells within the corresponding cluster. *FKBP10* expression patterns were further examined using cluster-level average expression and the proportion of expressing cells. *FKBP10* expression across malignant, stromal, and immune compartments was summarized by the average normalized expression level and the proportion of *FKBP10*-expressing cells in each cell population.

### 2.10 Gene Set Enrichment Analysis (GSEA)

To elucidate the biological pathways and potential mechanisms underlying the risk stratification, Genes were ranked by log2 fold change between the high- and low-risk groups. Enrichment was assessed using 10,000 permutations with a weighted enrichment statistic (gseaParam = 1). Gene sets containing 15–500 genes were tested, and pathways with Benjamini–Hochberg-adjusted FDR < 0.05 were considered significantly enriched. Based on the calculated risk scores, patients were categorized into high- and low-risk groups. KEGG pathway GSEA was performed using the clusterProfiler R package with gene sets obtained from the Kyoto Encyclopedia of Genes and Genomes (KEGG) database. Genes were ranked by log_2_ fold change between the high- and low-risk groups. Pathways with |NES| > 1, nominal *p* < 0.05, and FDR < 0.05 were considered significantly enriched. Specifically, the enrichment of pathways associated with tumor metastasis, extracellular matrix (ECM) remodeling, and neural physiological functions was evaluated. The GSEA results were visualized using enrichment plots, illustrating the running enrichment scores (RES) and the distribution of gene members within the ranked gene list for each representative pathway.

### 2.11 Gene Co-Expression and Correlation Analysis

To examine the association of *FKBP10* with EMT- and ECM-related genes, co-expression analysis was performed in the GSE49710 cohort (*n* = 498, GPL16876). Clinical information included age, sex, MYCN status, INSS stage, high-risk status, disease progression, and survival outcomes. Normalized, log_2_-transformed expression data were obtained from GEO, and probe identifiers were mapped to gene symbols before analysis. Correlation results were interpreted as gene-expression associations rather than evidence of direct regulation. The correlation between *FKBP10* mRNA expression and canonical markers of the epithelial-mesenchymal transition (EMT) (e.g., *CDH2*, *SNAI2*, *FN1*, *TWIST1*, *VIM*, and *ZEB1*), as well as extracellular matrix (ECM) remodeling genes (e.g., *LUM*, *COL1A1*, *ACTA2*, *COL1A2*, *COL3A1*, and *DCN*) was assessed using Pearson correlation analysis. The results were visualized via scatter plots featuring linear regression fits (red dashed lines) and 95% CI (gray shaded areas). Pearson correlation coefficients were calculated between *FKBP10* and EMT/ECM-related genes. *p*-values were adjusted for multiple testing using the Benjamini–Hochberg method, with an FDR < 0.05 considered statistically significant.

### 2.12 Cell Culture

Human neuroblastoma cell lines, including IMR-32 (CTCC-900-0802), SK-N-DZ (CTCC-001-0225), SK-N-BE(2) (CTCC-003-0110), SH-SY5Y (CTCC-001-0040), SK-N-AS (CTCC-003-0109), and SK-N-SH (CTCC-001-0015) were purchased from Meisen Cell Technology Co., Ltd. (Zhejiang, China). All cells were cultured in Dulbecco’s modified Eagle’s medium (DMEM, Gibco, Thermo Fisher Scientific, Cat: 6125473, Waltham, MA, USA) and Dulbecco’s Modified Eagle’s Medium/Nutrient Mixture F-12(Ham) (DMEM/F-12, Gibco, Thermo Fisher Scientific, Cat: 6125517, Waltham, MA, USA). The medium was supplemented with 10% heat-inactivated fetal bovine serum (FBS, Pricella, Cat: 164210-50, Wuhan, China) and 1% penicillin/streptomycin (Pricella, Cat: PB180120, Wuhan, China). Cells were maintained at 37°C in a humidified incubator with 5% CO_2_. All cell lines were authenticated by short tandem repeat (STR) profiling and confirmed to be free of mycoplasma contamination prior to use in this study.

### 2.13 Lentiviral Packaging and Generation of Stable Cell Lines

Lentiviral *sh-FKBP10#1*, *sh-FKBP10#2*, non-targeting negative control (NC), *FKBP10* overexpression (*OE-FKBP10*), and corresponding empty-vector constructs were generated by Tsingke Biotechnology (Beijing, China). shRNA constructs used the pLKO.1-puro backbone, with target sequences: sh-*FKBP10*#1: 5′-AAGGAAGTGGTTTAGGGATGG-3′; sh-*FKBP10*#2: 5′-ATATAGGTATTGTAGGTGTGG-3′. For *FKBP10* overexpression, human full-length *FKBP10* CDS was cloned into the TK-PCDH-copGFP-T2A-Puro lentiviral backbone without extra protein tags. The overexpression construct was generated through a commercial custom synthesis service. To produce recombinant lentivirus, HEK-293T cells were seeded at 6 × 10^6^ per 10 cm dish 24 h before transfection. Target plasmids were co-transfected with packaging plasmids psPAX2 and pMD2.G (mass ratio 4:3:1) using PEI (Beyotime, Cat: C0537, Shanghai, China) at a PEI-to-total-DNA mass ratio of 3:1. Transfection complexes were incubated at room temperature for 20 min before addition to culture medium. Viral supernatants were collected at 48 h and 72 h post-transfection, clarified by centrifugation, filtered through 0.45 μm membranes, and titrated via limiting-dilution puromycin-selection in HEK-293T cells. For stable knockdown, SK-N-BE(2) and SH-SY5Y cells were transduced once at MOI = 10 with filtered lentivirus plus 8 μg/mL Polybrene (Beyotime, Cat: C0351, Shanghai, China). After 48 h, cells were maintained in 2 μg/mL puromycin (Beyotime, Cat: ST551, Shanghai, China), with medium refreshed routinely until all untransduced control cells had died. IMR-32 cells were transduced with OE-*FKBP10* or empty-vector lentivirus under identical conditions for stable overexpression. Western blot was used to verify *FKBP10* knockdown and overexpression efficiency in the resulting stable cell lines.

### 2.14 Cell Proliferation and Viability Assay

Cell proliferation kinetics were determined via the Cell Counting Kit-8 (CCK-8) assay. SK-N-BE(2) and SH-SY5Y (NC/*FKBP10* knockdown), as well as IMR-32 (empty vector/*FKBP10* overexpression) cells were seeded in 96-well plates at 3000 cells/well and cultured under humidified 37°C, 5% CO_2_ conditions. Three independent biological replicates with six technical replicates per condition were included. At 0, 24, 48, and 72 h post-adhesion, 10 μL CCK-8 reagent (Solarbio, Cat: CA1210, Beijing, China) was added to each well, followed by a 3-h incubation. Absorbance at 450 nm was read on a microplate reader (Synergy H1, BioTek, Winooski, VT, USA). After subtracting blank well background, relative proliferation was calculated by normalizing corrected OD values of knockdown groups to the NC group.

For drug sensitivity assays, SH-SY5Y NC and SH-SY5Y sh-*FKBP10*#1 cells were plated at 3000 cells/well in 96-well plates and incubated overnight for attachment. Cells were exposed to serially diluted MK-2206 (Aladdin, Cat: 1032350 13 2, Shanghai, China), lapatinib (Aladdin, Cat: L126696, Shanghai, China), or cisplatin (Aladdin, Cat: C295225, Shanghai, China) for 24 h. DMSO (Solarbio, Cat: D8372, Beijing, China) was used as vehicle control; the final DMSO concentration across all groups was kept ≤ 0.1% (*v*/*v*) to exclude solvent-induced cytotoxicity. Each condition contained three biological replicates and six technical replicates. After treatment, 10 μL of CCK-8 solution was added, and plates were incubated for another 3 h at 37°C. OD values at 450 nm were measured using the same Synergy H1 microplate reader. Cell viability was computed after background subtraction and normalized to untreated controls. Dose-response curves were plotted, and IC_50_ values were calculated by nonlinear regression analysis.

### 2.15 Clonogenic Assay

To evaluate long-term proliferative and clonogenic capacity, a clonogenic assay was performed. Briefly, single-cell suspensions of control (NC), *FKBP10*-knockdown, stable *FKBP10*-overexpressing IMR-32 and corresponding vector control cells, as well as *FKBP10*-rescued SH-SY5Y and SK-N-BE(2) cells were prepared and seeded into 6-well plates at 1000 cells per well. For rescue experiments, shRNA-resistant *FKBP10* overexpression plasmids were transfected into *FKBP10*-knockdown cells to avoid shRNA-mediated silencing. Restored *FKBP10* protein expression was verified by Western blotting before seeding to ensure experimental reliability. Following a one-week culture period to allow for colony formation, the cells were fixed with 4% paraformaldehyde (Biosharp, Cat: G0528, Hefei, China) for 15 min at room temperature and subsequently stained with 0.1% crystal violet (Solarbio, Cat: G1063, Beijing, China) for 15 min. The plates were carefully washed with distilled water to remove excess stain and allowed to air dry. For accurate quantification, viable colonies, defined as discrete cell clusters containing over 50 cells, were manually counted using an inverted microscope (CKX41, Olympus, Tokyo, Japan).

### 2.16 EdU Cell Proliferation Assay

Cell proliferation was assessed using the BeyoClick™ EdU Cell Proliferation Kit with Alexa Fluor 488 (Beyotime, Shanghai, China). The cell groups assessed stable *FKBP10* knockdown and control cells, stable *FKBP10*-overexpressing IMR-32 cells and matched vector control cells, and *FKBP10* rescue model cells. For the rescue model, *FKBP10*-knockdown SH-SY5Y and SK-N-BE(2) cells were transfected with *FKBP10* overexpression plasmids to restore *FKBP10* expression, and successful protein restoration was validated by Western blotting prior to the EdU assay. All cells were incubated with 10 μM EdU for 4 h at 37°C. Subsequently, the cells were fixed with 4% paraformaldehyde (Biosharp, Cat: G0528, Hefei, China), permeabilized with 0.3% Triton X-100 (Nuoning Biotechnology, Cat: NBS0442, Shanghai, China) and subjected to the Click reaction for 30 min in the dark according to the manufacturer’s protocol. Nuclei were counterstained with DAPI. Fluorescence images were acquired using a Leica TCS SP8 confocal microscope (Leica Microsystems, Wetzlar, Germany). All assays were performed in three biological replicates. For quantification, five random non-overlapping fields with uniform and artifact-free cell distribution were selected per sample, with a minimum of 200 cells analyzed per group. The proliferation rate was determined via ImageJ software (v1.54f, National Institutes of Health, Bethesda, MD, USA) by calculating the ratio of EdU-positive cells to total DAPI-stained nuclei.

### 2.17 Wound Healing Assay

To evaluate two-dimensional cell migration, a wound healing assay was performed. Neuroblastoma cells were seeded into 6-well plates at an initial density of 1 × 10^6^ cells per well and cultured for 24 h to form a monolayer. Only cell monolayers reaching 100% uniform confluence without gaps or cell aggregation were used for consistent scratch wounding and subsequent wound-healing measurements. A linear scratch wound was artificially created across the center of each well using a sterile 10 μL pipette tip. Detached cells and debris were subsequently removed by washing the wells gently twice with PBS. Cell migration into the denuded area was monitored, and representative images were captured at 0 and 24 h post-wounding using an inverted microscope (CKX31, Olympus, Tokyo, Japan). To eliminate proliferative interference, cells were cultured in serum-free medium during the 24-h assay period. For each sample, five random microscopic fields were analyzed. The wound closure rate was calculated via ImageJ software (v1.54f, NIH) using the formula: Wound healing percentage (%) = (Initial 0 h wound area − 24 h residual wound area)/Initial 0 h wound area × 100%. All assays were conducted with three biological and three technical replicates to ensure reproducibility.

### 2.18 Transwell Migration and Invasion Assays

Cell migration and invasion were assessed using 24-well Transwell inserts (8.0 μm pore size; Corning, Cat: 3422, Tewksbury, MA, USA). For invasion assays, inserts were pre-coated with Matrigel. Matrigel was thawed overnight at 4°C, diluted to 1 mg/mL in serum-free medium, and 50 μL of diluted Matrigel was loaded per insert, followed by a 2-h incubation at 37°C, 5% CO_2_ for gel polymerization before cell seeding. Uncoated inserts were used for migration assays. Stable *FKBP10-*knockdown and control cells, *FKBP10-*overexpressing IMR-32 and empty-vector control cells, and *FKBP10-*rescue SH-SY5Y and SK-N-BE(2) cells were used. Successful *FKBP10* re-expression in rescue cell lines was pre-validated by Western blot prior to functional assays. 5 × 10^4^ cells suspended in 200 μL serum-free medium were seeded into upper chambers; lower chambers contained medium supplemented with 10% FBS as a chemoattractant. After 48 h of incubation, cells remaining on the upper membrane surface were wiped off with sterile cotton swabs. Cells that had crossed the membrane were fixed in methanol and stained with 0.1% crystal violet. Each experiment included three biological replicates with three technical replicates per condition. For quantification, five non-overlapping random fields were imaged per insert using an Olympus BX53 microscope (Tokyo, Japan) to eliminate regional bias. Blinded manual counting was performed to quantify migrated and invaded cells.

### 2.19 Western Blot Analysis

Total cellular proteins were extracted from the cultured neuroblastoma cells using RIPA lysis buffer (Sigma-Aldrich, Cat: R0278, ST. Louis, MO, USA) supplemented with protease and phosphatase inhibitors (100×, Yeasen Biotechnology, Cat: 20124ES03, Shanghai, China). Protein concentrations were determined using a BCA protein assay kit (Beyotime, Cat: P0010, Shanghai, China). Equal amounts of total protein (30 μg per lane) were resolved by 10% sodium dodecyl sulfate-polyacrylamide gel electrophoresis (SDS-PAGE) and subsequently transferred onto polyvinylidene fluoride (PVDF) membranes. To prevent non-specific binding, the membranes were blocked with 5% non-fat milk in Tris-buffered saline containing 0.1% Tween-20 (TBST) for 1 h at room temperature. The membranes were then incubated overnight at 4°C with the following primary antibodies: anti-FKBP10 (1:1000, Cat. 12172-1-AP), anti-MMP2 (1:1000, Cat. 10373-2-AP), anti-MMP9 (1:1000, Cat. 10375-2-AP), anti-E-cadherin (1:1000, Cat. 20874-1-AP), anti-N-cadherin (1:1000, Cat. 22018-1-AP), and anti-vimentin (1:1000, Cat. 10366-1-AP), all raised in rabbit and purchased from Proteintech (Wuhan, China). The *FKBP10* antibody was experimentally validated for specific reactivity with both human and mouse samples via Western blotting, and no off-target bands were observed in our experimental system, confirming its high specificity for endogenous *FKBP10* protein detection. Anti-β-actin antibody (1:5000, Cat. T0004, mouse monoclonal, Affinity Biosciences, Changzhou, China) was used as the internal loading control. After three washes with TBST, the membranes were incubated with the corresponding horseradish peroxidase (HRP)-conjugated secondary antibodies for 1 h at room temperature. Depending on the host species of the primary antibodies, HRP-linked goat anti-rabbit IgG (1:5000, Cat. SA00001-2, Proteintech, Wuhan, China) and HRP-linked goat anti-mouse IgG (1:5000, Cat. SA00001-1-A, Proteintech, Wuhan, China) were used as secondary antibodies. Following rigorous washing, the immunoreactive protein bands were visualized using an enhanced chemiluminescence (ECL) reagent kit (Yeasen Biotechnology, Cat: 36222ES60, Shanghai, China). The chemiluminescent signals were captured using an eBlot Touch Imager (Shanghai, China). All Western blot experiments were performed with three independent biological replicates. Representative blot images are presented in the figures. Band densitometry was analyzed using ImageJ software (v1.54f, NIH). The relative protein expression levels were normalized to the internal reference β-actin.

### 2.20 In Vivo Tumor Xenograft Model

All animal experiments were approved by the Animal Ethics Committee of Tianjin Medical University Cancer Institute and Hospital, China, under approval number AE-2025011. Fifteen female BALB/c nude mice (4–6 weeks, 18–22 g) were purchased from Vital River Laboratory Animal Technology Co., Ltd. All mice were maintained under specific pathogen-free conditions (12 h light/dark cycle, 22 ± 2°C, 50 ± 10% humidity) with free access to standard diet and sterile water. After 7 days of acclimatization, mice were randomly grouped, and blinding was performed during experiments and data analysis. All animal procedures were performed in accordance with the ARRIVE Essential 10 guidelines, with no animal exclusion during analysis.

The mice were randomly assigned to three groups: NC, *sh-FKBP10#1*, and *sh-FKBP10#2*, with five mice in each group. SH-SY5Y cells in the logarithmic growth phase were harvested, and cell viability was confirmed to be greater than 90% before injection. A total of 2 × 10^7^ viable stable *FKBP10*-knockdown or control SH-SY5Y cells suspended in 100 μL PBS were subcutaneously injected into the right inguinal region of each mouse. Tumor length and width were measured every 5 days using a digital caliper, and tumor volume was calculated as V = 0.5 × length × width^2^. On day 28 after injection, all mice were euthanized via CO_2_ inhalation, and tumors were excised and weighed; tumor growth curves were plotted.

### 2.21 Statistical Analysis

Statistical analyses were performed using R software version 4.2.2 (R Foundation for Statistical Computing, Vienna, Austria) and GraphPad Prism version 10.1.2 (GraphPad Software, San Diego, CA, USA). Continuous variables are presented as mean ± standard deviation or median with interquartile range, as appropriate. Differences between two groups were assessed using Student’s *t*-test or the Mann-Whitney U test, depending on data distribution. Comparisons among multiple groups were performed using one-way analysis of variance followed by appropriate *post hoc* tests or the Kruskal-Wallis test. Overall survival was analyzed using the Kaplan-Meier method, and differences between survival curves were evaluated using the log-rank test. Cox proportional hazards regression analysis was used to estimate HRs and 95% CIs. Correlations between risk scores and tumor microenvironment-related features were assessed using Spearman’s rank correlation analysis. For multiple comparisons, false discovery rate correction was applied where appropriate. Tumor growth curves in xenograft experiments were compared using repeated-measures ANOVA or mixed-effects models. A two-sided *p* value < 0.05 was considered statistically significant.

## 3 Results

### 3.1 Identification of FKBP10 as a CAF-Related Prognostic Biomarker for Neuroblastoma via Multi-Cohort Bioinformatic Analysis

Given the critical role of the tumor microenvironment in NB progression, we hypothesized that CAF infiltration may affect NB prognosis. Herein, we evaluated the clinical prognostic relevance of CAF infiltration using public transcriptomic datasets. CAF abundance was quantified via the xCell algorithm, and patients were stratified into high- and low-CAF groups for survival comparison. In the GSE49710 cohort, elevated CAF infiltration was associated with shorter OS (log-rank *p* = 0.002, HR = 2.787, 95% CI: 1.400–5.551). The independent E-MTAB cohort further supported this trend, showing that higher CAF abundance was correlated with inferior OS (Fig. 1A; log-rank *p* = 0.001, HR = 2.100, 95% CI: 1.322–3.336). Collectively, these results suggest that increased CAF infiltration is likely associated with adverse clinical outcomes in NB.

Having observed the prognostic implication of CAF infiltration in NB, we next sought to explore potential core transcriptional networks linked to CAF-rich stromal remodeling using weighted gene co-expression network analysis (WGCNA) across four independent NB cohorts (TARGET, GSE45547, GSE49710, and E-MTAB). Appropriate soft-thresholding powers were determined to construct scale-free co-expression networks for each dataset. To improve the reliability of stromal feature characterization, multiple deconvolution algorithms (EPIC, MCPcounter, xCell) and the overall StromalScore were integrated to quantify CAF and stromal signatures. Module–trait correlation analysis identified several gene modules that tend to be consistently and positively correlated with stromal and CAF phenotypes across cohorts (Fig. 1B and Fig. S1). Specifically, the pink module in the TARGET cohort showed a strong tendency toward positive correlation with CAF infiltration (R = 0.88, *p* = 1 × 10^−83^). The green module in GSE45547 (R = 0.68, *p* = 6 × 10^−16^), the green-yellow module in GSE49710 (R = 0.90, *p* = 3 × 10^−183^), and the magenta and purple modules in E-MTAB also exhibited prominent stroma-related correlation patterns. All heatmap cells represent Pearson correlation coefficients and corresponding *p* values, with red and blue indicating positive and negative correlations, respectively. These findings imply that conserved co-expression modules may underlie CAF-associated stromal reprogramming in NB.

To screen potentially stable and high-confidence CAF-related genes, we intersected module genes derived from the four WGCNA datasets, yielding 75 consensus CAF signature genes for subsequent filtering (Fig. 1C). We further constructed a multi-model machine learning framework (GLM, RF, SVM, XGBoost) to explore the potential predictive capacity of these candidate genes for stromal/CAF characteristics. Residual analysis demonstrated that the SVM and XGB models exhibited relatively lower RMSE and comparatively better overall fit (Fig. 1D). ROC validation further confirmed the relatively favorable discriminatory capacity of the SVM model (AUC = 0.938), compared with RF (AUC = 0.915), XGB (AUC = 0.891), and GLM (AUC = 0.786) (Fig. 1E). Accordingly, the SVM model was considered suitable for subsequent feature gene prioritization.

Feature importance ranking based on permutation RMSE loss identified several genes that tended to rank highly across machine learning models, including VCAN, MFAP4, and *FKBP10* (Fig. 1F). Notably, these genes achieved relatively high weights in the SVM model, suggesting their potential relevance to CAF stromal traits in NB. We further performed survival validation for the top SVM features. Univariate Cox regression analysis of the top 20 SVM-ranked genes revealed that only *FKBP10*, *MFAP4*, and *VCAN* exhibited significant correlations with OS in NB patients (Fig. 1G). *FKBP10* showed the most prominent risk-related correlation (HR = 2.162, 95% CI: 1.446–3.233, *p* < 0.001), followed by *MFAP4* (HR = 1.675, 95% CI: 1.230–2.281, *p* = 0.001) and *VCAN* (HR = 1.459, 95% CI: 1.051–2.026, *p* = 0.024). To reduce gene redundancy and develop a relatively concise CAF-associated prognostic signature, the three genes were incorporated into a *LASSO-Cox* regression model. The optimal lambda value was determined via 10-fold cross-validation (Fig. 1H,I). Ultimately, *VCAN* was excluded, while *FKBP10* and *MFAP4* retained non-zero coefficients (Fig. 1J). Importantly, *FKBP10* showed a noticeably higher regression coefficient than *MFAP4*, implying its potentially dominant contribution to the CAF-related prognostic signature.

In summary, through the stepwise integration of survival analysis, multi-cohort WGCNA, cross-dataset gene intersection, machine learning screening, and prognostic modeling, our study indicates that high CAF infiltration is likely associated with unfavorable prognosis in NB and suggests that *FKBP10* is a key candidate gene linked to CAF-related tumor progression, providing a rationale for further experimental validation of the biological functions of *FKBP10* in NB stromal remodeling and malignant progression.

**Figure 1:**
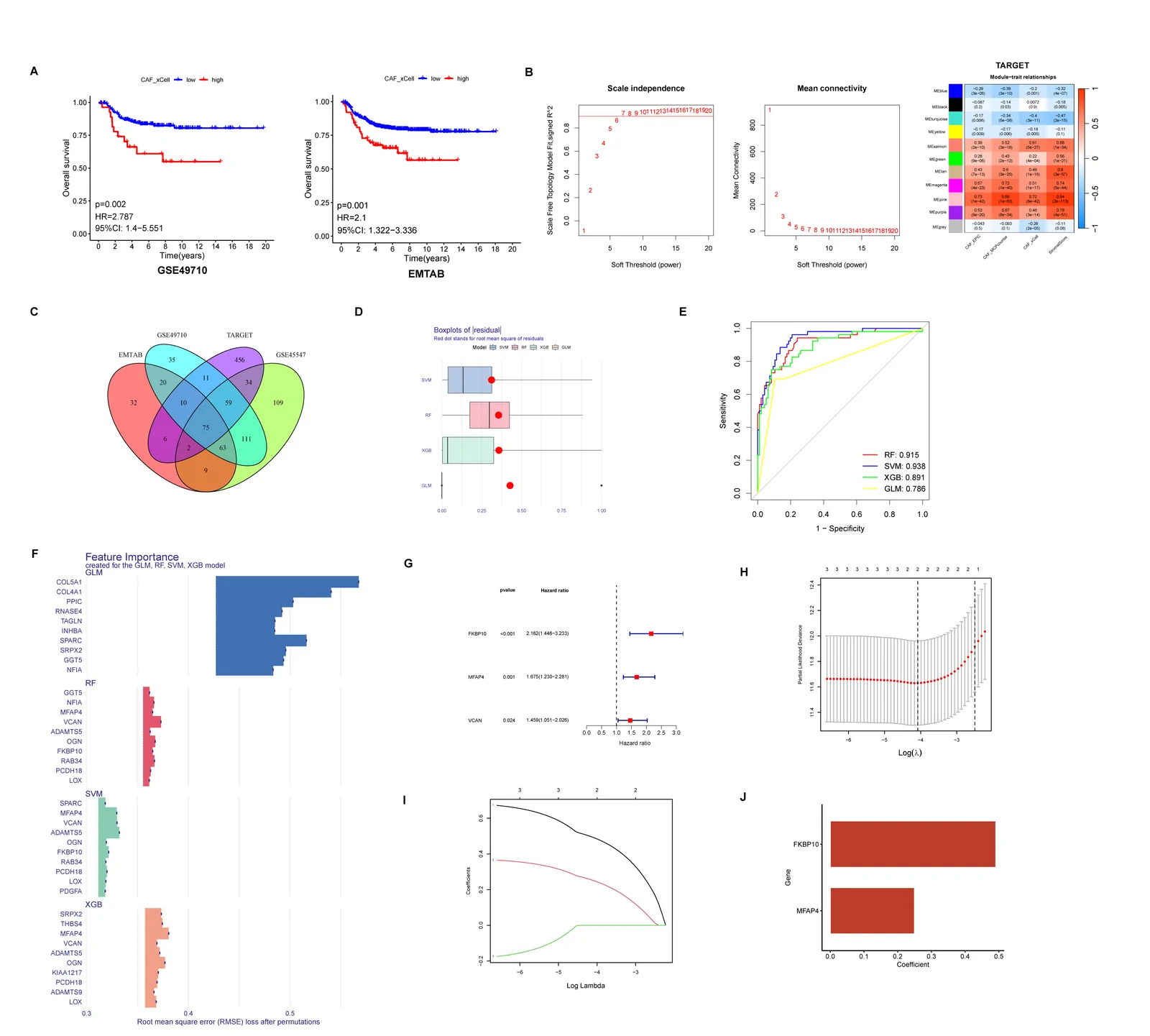
Identification of CAF-associated prognostic features and construction of an *FKBP10*-dominant risk model in neuroblastoma. (**A**) Kaplan–Meier survival analysis of overall survival stratified by CAF infiltration levels in the GSE49710 and E-MTAB cohorts. (**B**) For the TARGET cohort, the left and middle panels show the scale-free topology fit index and mean connectivity across different soft-thresholding powers, and the right panel shows the module–trait relationship heatmap. In the heatmap, rows represent module eigengenes, and columns represent the stromal-related traits estimated by EPIC, MCPcounter, xCell, and StromalScore. Values in each cell indicate the Pearson’s correlation coefficient and the corresponding *p* value; red denotes positive correlation, and blue denotes negative correlation. (**C**) Venn diagram illustrating the overlapping CAF-related genes from four independent WGCNA datasets (E-MTAB, GSE49710, TARGET, GSE45547), yielding 75 consensus candidate genes. (**D**) Boxplots showing the absolute residual distribution of four machine learning models (SVM, RF, GLM, XGB), with red dots representing the root mean square error (RMSE) of each model. (**E**) Receiver operating characteristic (ROC) curves comparing the predictive efficacy of the four machine learning algorithms, with corresponding area under the curve (AUC) values displayed. (**F**) Variable importance ranking of candidate genes calculated by permutation-based RMSE loss across four machine learning models. (**G**) Forest plot of univariate Cox regression analysis showing the prognostic significance of three core candidate genes (*FKBP10*, *MFAP4*, *VCAN*), with hazard ratios (HRs) and 95% confidence intervals (CIs) summarized. (**H**) 10-fold cross-validation for optimal λ parameter selection in the *LASSO-Cox* model based on partial likelihood deviance. Vertical dashed lines indicate the optimal λ value. (**I**) *LASSO* coefficient curves of candidate genes along the log(λ) gradient. (**J**) Bar plot of non-zero coefficients for genes included in the final *LASSO* prognostic model, showing *FKBP10* as the dominant contributor.

### 3.2 Single-Cell Transcriptomic Analysis Reveals Distinct FKBP10 Distribution in Tumor Stromal and Immune Microenvironment of Neuroblastoma

To further dissect the cellular localization and biological implication of the CAF-associated risk signature, we characterized the NB TME at single-cell resolution. After rigorous quality control (QC) and dataset integration, single-cell RNA-seq QC metrics were systematically evaluated (Fig. 2A–C), including the number of detected genes per cell (nFeature_RNA, A), total UMI counts per cell (nCount_RNA, B), and mitochondrial transcript percentage (percent.mt, C) across all samples. Representative lineage markers were used to annotate major cell populations, encompassing malignant NB cells, fibroblasts, macrophages, T cells, endothelial cells, and other non-malignant stromal and immune subsets (Fig. 2D), laying a foundation for subsequent localization analysis of the risk signature.

With cell-type annotation completed, we next performed AUCell analysis to quantify the single-cell-level enrichment of the previously identified CAF-related prognostic signature across diverse TME compartments. AUCell-based single-cell enrichment analysis revealed that the CAF-related risk signature was preferentially enriched in fibroblast clusters, particularly cluster 22, while most immune cell subsets exhibited relatively low signature enrichment (Fig. 2E). These single-cell patterns were consistent with the bulk transcriptomic results obtained in the previous section, indicating that the risk signature is primarily correlated with fibroblast-dominated stromal features within the NB TME, without ruling out potential contributions from other cellular components.

Given the stromal tendency of the multi-gene signature, we further focused on the core prognostic gene *FKBP10* to clarify its *in situ* expression distribution. UMAP feature plots showed that *FKBP10* expression was sparsely distributed and restricted to specific cell subsets in the TME (Fig. 2F,G). Further quantification of *FKBP10*-positive cells across annotated cell populations demonstrated distinct expression patterns between the overall signature and its core gene (Fig. 2H). While the signature was largely confined to fibroblast populations, *FKBP10* exhibited a broader expression spectrum. Beyond canonical stromal fibroblasts and partial myeloid immune cells, a considerable proportion of malignant neuroblastoma cells also exhibited detectable *FKBP10* expression.

Collectively, single-cell transcriptomic profiling refined our bulk-level observations. The CAF-derived prognostic signature preferentially reflects stromal characteristics of fibroblast compartments in the TME. This heterogeneous expression feature suggests that *FKBP10* is not merely a stromal indicator but may also correlate with both TME remodeling and tumor cell-intrinsic properties.

**Figure 2:**
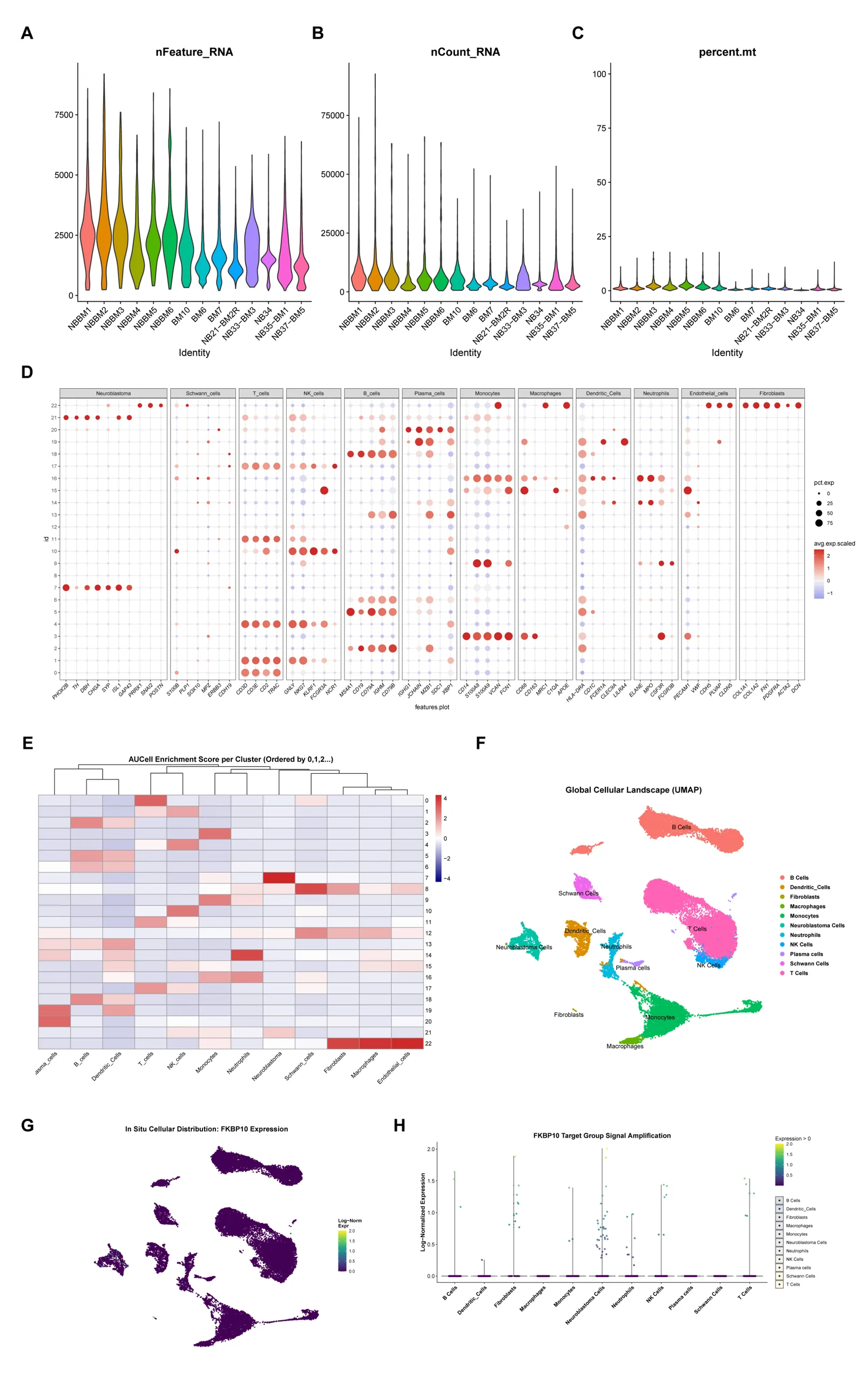
Single-cell transcriptomic characterization of the neuroblastoma tumor microenvironment and cellular localization of the risk signature and *FKBP10*. (**A**–**C**) Quality-control distributions of single-cell RNA-seq data across samples, showing the number of detected genes per cell (**A**, nFeature_RNA), total UMI counts per cell (**B**, nCount_RNA), and mitochondrial transcript percentage (**C**, percent.mt). (**D**) Dot plot of canonical marker genes used for cell-type annotation across Seurat clusters. Dot size indicates the proportion of expressing cells, and color intensity represents the average scaled expression level. (**E**) Heatmap of AUCell enrichment scores for the established risk signature across annotated cell clusters, showing preferential enrichment in fibroblast-related populations. (**F**) UMAP projection of the integrated single-cell dataset, with cells colored by annotated cell type. (**G**) UMAP feature plot showing the distribution and relative expression of *FKBP10* across the single-cell landscape. (**H**) Distribution of *FKBP10*-positive cells across annotated cell types. Each dot represents a cell with detectable *FKBP10* expression, and the *y*-axis indicates log-normalized expression level.

### 3.3 FKBP10-Associated CAF Signature Predicts Prognosis, Stromal Activation, Immune Evasion and Therapeutic Resistance in Neuroblastoma

After establishing a *LASSO*-derived prognostic signature primarily weighted by *FKBP10*, we next evaluated its prognostic performance and biological relevance in NB. Based on the calculated risk scores, patients were stratified into high- and low-risk groups, and Kaplan–Meier survival analysis was performed. In the primary cohort, patients classified as high-risk showed significantly worse OS than those in the low-risk group (Fig. 3A; HR = 1.982, 95% CI: 1.329–2.956, *p* < 0.001). A similar survival pattern was observed in the independent validation cohort (Fig. 3B; HR = 2.784, 95% CI: 1.425–5.439, *p* = 0.002), consistent with reproducible prognostic stratification.

Because this signature was derived from CAF-associated co-expression modules, we examined whether the resulting risk score was correlated with stromal characteristics within the tumor microenvironment. Correlation analyses were performed between the risk score and multiple established stromal/CAF estimation metrics, including EPIC, MCPcounter, xCell, ESTIMATE-derived StromalScore, and TIDE-related stromal indices. In the primary cohort, the risk score showed consistently positive correlations with CAF- and stroma-related measures (Fig. 3C), including strong correlations with CAF_EPIC, CAF_xCell, and StromalScore. Similar correlation patterns were observed in the validation cohort (Fig. 3D). Collectively, these findings suggest that the *FKBP10*-dominant risk signature is not only associated with prognosis but also correlated with an activated stromal state characterized by increased CAF infiltration.

Given the reported association between CAF-rich stroma and immune suppression as well as immune checkpoint blockade (ICB) resistance, we next explored whether this CAF-related risk signature was associated with predicted immunotherapy response. The predicted proportion of ICB non-responders was markedly higher in the high-risk group than in the low-risk group (86% vs. 53%, *p* < 0.001; Fig. 3E). Receiver operating characteristic (ROC) analysis further showed that the risk model had a moderate discriminatory capacity for predicted immunotherapy response, with an area under the curve (AUC) of 0.765 (95% CI: 0.695–0.829; Fig. 3F). Using the Tumor Immune Dysfunction and Exclusion (TIDE) framework, we found that the high-risk group had significantly higher TIDE scores than the low-risk group (Fig. 3G; *p* < 0.001), consistent with a greater likelihood of immune evasion and T-cell exclusion. These results suggest that the stromal risk signature may help identify patients with a lower predicted likelihood of immunotherapy benefit, although clinical validation in immunotherapy-treated cohorts remains warranted.

We then investigated whether this high-risk stromal phenotype was also associated with altered predicted sensitivity to conventional anticancer and targeted agents. Drug response prediction analyses showed that patients in the high-risk group tended to have lower estimated sensitivity scores for several commonly used chemotherapeutic agents, including cisplatin (*p* = 3.7 × 10^−5^), 5-fluorouracil (*p* = 8.1 × 10^−10^), topotecan (*p* = 0.00025), and irinotecan (*p* = 8.3 × 10^−7^). Lower predicted sensitivity was also observed for selected targeted agents, including lapatinib (*p* = 2.1 × 10^−5^) and the AKT inhibitor MK-2206 (*p* = 5 × 10^−6^), in the high-risk group relative to the low-risk group (Fig. 3H).

Taken together, these analyses support the robustness of the *FKBP10*-dominant CAF-related signature for prognostic stratification and further suggest its association with a biologically relevant stromal context in NB. Moreover, the high-risk phenotype defined by this signature was correlated with predicted immune evasion, a reduced predicted likelihood of immunotherapy benefit, and decreased estimated sensitivity to multiple chemotherapeutic and targeted agents, suggesting an association between CAF-enriched stromal programs and both adverse prognosis and therapeutic resistance.

**Figure 3:**
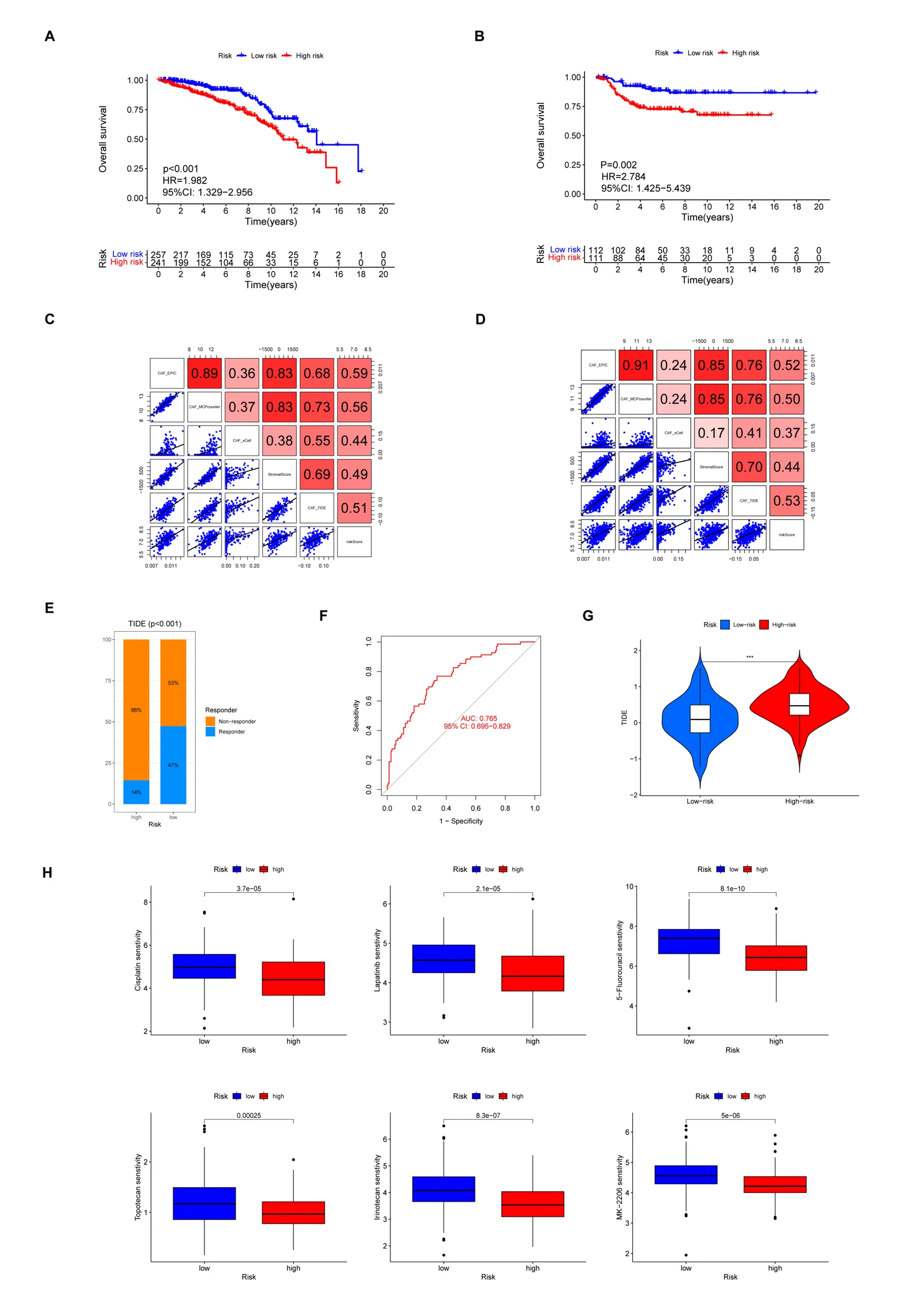
Prognostic, microenvironmental, and therapeutic relevance of the CAF-related risk signature in neuroblastoma. (**A**,**B**) Kaplan–Meier curves for overall survival comparing high-risk and low-risk patients in the training cohort (**A**) and validation cohort (**B**). *p* values were determined by the log-rank test, and numbers at risk are shown below the curves. (**C**,**D**) Correlation matrices showing the associations between the risk score and stromal/CAF-related infiltration metrics estimated by multiple algorithms in the training cohort (**C**) and validation cohort (**D**). Values indicate correlation coefficients. (**E**) Stacked bar plot comparing the proportions of predicted immunotherapy responders and non-responders between the high-risk and low-risk groups based on TIDE classification. (**F**) Receiver operating characteristic (ROC) curve evaluating the performance of the risk signature in predicting immunotherapy response. The AUC and 95% CI are indicated. (**G**) Violin plot showing the distribution of TIDE scores in the low-risk and high-risk groups. (**H**) Boxplots comparing the predicted drug sensitivity between risk groups for cisplatin, lapatinib, 5-fluorouracil, topotecan, irinotecan, and MK-2206. ***p < 0.001. AUC, area under the curve, HR, hazard ratio, CI, confidence interval.

### 3.4 FKBP10 Expression and Genetic Manipulation Correlate with In-Vitro Proliferative Phenotypes of Neuroblastoma Cells

Given the single-cell transcriptomic observation that *FKBP10* is expressed in malignant NB cells beyond stromal compartments, we further characterized endogenous *FKBP10* expression and its phenotypic correlation in cultured NB cells. Western blotting was used to detect basal *FKBP10* protein levels in six human NB cell lines (IMR-32, SK-N-DZ, SK-N-BE(2), SH-SY5Y, SK-N-AS, SK-N-SH). *FKBP10* protein was detectable across all tested lines with variable expression levels (Fig. 4A). SK-N-BE(2) and SH-SY5Y were selected for loss-of-function studies given their relatively high *FKBP10* abundance; IMR-32 was used for complementary gain-of-function experiments. Two independent lentiviral shRNAs (*sh-FKBP10#1*, *sh-FKBP10#2*) were used to generate stable *FKBP10*-silenced SK-N-BE(2) and SH-SY5Y cell lines, with scrambled shRNA as a negative control (NC). An *FKBP10*-overexpressing IMR-32 cell model was also constructed. Western blot verified efficient *FKBP10* knockdown in shRNA groups and elevated ectopic *FKBP10* in IMR-32 overexpression cells (Fig. 4B), confirming successful model construction for subsequent phenotypic assays. CCK-8 assays showed reduced cell growth over time in *FKBP10*-knockdown SK-N-BE(2) and SH-SY5Y compared with NC, whereas *FKBP10* overexpression in IMR-32 was associated with higher cell viability (Fig. 4C). Colony-formation assays recapitulated these tendencies: *FKBP10* silencing correlated with decreased clonogenic capacity, and *FKBP10* overexpression correlated with increased colony numbers (Fig. 4D,E). The extent of phenotypic change differed among cell lines and constructs, yet the overall observations aligned with a growth-supporting association of *FKBP10* in NB cells. EdU incorporation assays were further performed to assess DNA-synthesis status. Representative fluorescent images and quantitative results are presented in Fig. 4F (EdU: green; DAPI: blue; scale bars shown). *FKBP10*-knockdown groups exhibited lower proportions of EdU-positive cells, while *FKBP10*-overexpressing IMR-32 displayed a higher EdU-positive fraction relative to controls. Combined CCK-8, colony-formation, and EdU data suggest that *FKBP10* may correlate with the proliferative and clonogenic potential of NB cells *in vitro*.

Collectively, these *in vitro* findings offer functional phenotypic evidence supporting prior bioinformatic and single-cell observations. *FKBP10* is endogenously expressed in NB cell lines, and genetic manipulation of *FKBP10* is associated with altered cell-growth-related phenotypes. *FKBP10* depletion correlates with reduced proliferation in SK-N-BE (2)/SH-SY5Y, and *FKBP10* overexpression correlates with enhanced proliferative phenotypes in IMR-32, consistent with *FKBP10* being potentially linked to pro-growth features in neuroblastoma.

To further confirm the causally relevant association between *FKBP10* and cell proliferation, rescue experiments were performed in *FKBP10*-silenced SK-N-BE(2) and SH-SY5Y cells by ectopic overexpression of *FKBP10* (*OE-FKBP10*). Western blot analysis validated the restoration of *FKBP10* protein levels following exogenous *FKBP10* overexpression in the *sh-FKBP10#1* background (Fig. S2A). Colony-formation assays revealed that re-introduction of *FKBP10* partially rescued the impaired clonogenic capacity induced by *FKBP10* knockdown (Fig. S2B). Consistently, EdU incorporation assays demonstrated that the decreased proportion of EdU-positive proliferative cells upon *FKBP10* silencing was partially restored after *FKBP10* re-expression (Fig. S2C). Collectively, these rescue experimental results further support the association between *FKBP10* abundance and the proliferative-clonogenic phenotypes of neuroblastoma cells *in vitro*.

To evaluate whether *FKBP10* status correlates with drug-response profiles in neuroblastoma cells, we performed cell viability assays to determine the half-maximal inhibitory concentration (IC_50_) of MK-2206, lapatinib, and cisplatin in SH-SY5Y-NC and SH-SY5Y-*sh-FKBP10#1* cells (Fig. S3A–F). Compared with control cells, *FKBP10*-silenced SH-SY5Y cells exhibited reduced IC_50_ values for MK-2206 (2.455 μM vs. 5.902 μM), lapatinib (2.844 μM vs. 4.634 μM), and cisplatin (5.225 μM vs. 8.002 μM). These data indicate that *FKBP10* depletion is associated with increased cellular sensitivity to AKT inhibitor MK-2206, tyrosine-kinase inhibitor lapatinib, and chemotherapeutic agent cisplatin in SH-SY5Y cells (Fig. S3).

**Figure 4:**
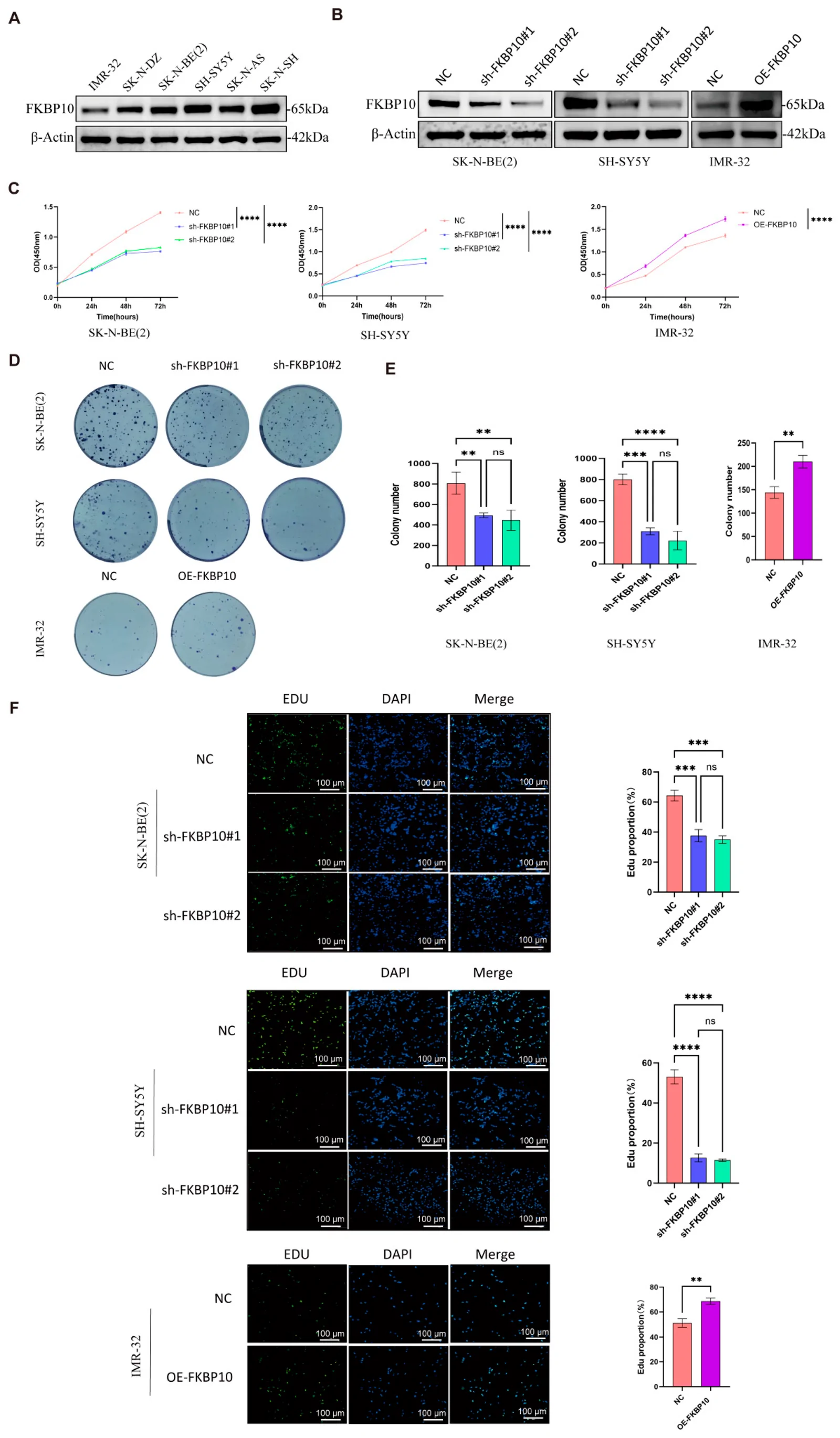
*FKBP10* expression profiling and functional validation of *FKBP10* perturbation in neuroblastoma cells. (**A**) Western blot analysis of endogenous *FKBP10* protein expression in six human neuroblastoma cell lines. β-actin served as the loading control. (**B**) Western blot validation of *FKBP10* knockdown in SK-N-BE(2) and SH-SY5Y cells transduced with two independent shRNAs (*sh-FKBP10#1* and *sh-FKBP10#2*), and *FKBP10* overexpression in IMR-32 cells. (**C**) CCK-8 assays showing cell viability over time following *FKBP10* knockdown in SK-N-BE(2) and SH-SY5Y cells or *FKBP10* overexpression in IMR-32 cells. (**D**) Representative images of colony formation assays in the indicated cell groups. (**E**) Quantification of colony numbers corresponding to panel D. (**F**) Representative EdU incorporation images and quantification of EdU-positive cells in the indicated groups. EdU-positive nuclei are shown in green and total nuclei are counterstained with DAPI (blue). Data are presented as mean ± standard deviation (SD) from three independent experiments. Statistical significance was determined by Student’s *t*-test or one-way ANOVA. **p < 0.01, ***p < 0.001, ****p < 0.0001; ns, not significant.

### 3.5 FKBP10 Silencing Restricts the Migratory and Invasive Capacities of Neuroblastoma Cells

Apart from supporting cell proliferation, metastatic dissemination represents a key biological feature of high-risk neuroblastoma. Having confirmed that *FKBP10* depletion suppresses cell growth, we further explored its potential association with tumor-cell migration and invasion. Wound-healing assays were first performed to assess two-dimensional (2D) migratory behavior. *FKBP10* knockdown in SK-N-BE(2) and SH-SY5Y cells delayed gap closure at 24 h, yielding a significantly lower wound-healing rate relative to negative-control (NC) cells (Fig. 5A). In contrast, ectopic *FKBP10* overexpression in IMR-32 cells was associated with faster wound closure than in the corresponding NC group, suggesting increased migratory behavior following *FKBP10* upregulation (Fig. 5A). Transwell assays were then used to validate these observations under transmembrane conditions. Consistent with the scratch-wound results, *FKBP10*-silenced cells displayed reduced vertical migratory potential. Likewise, *FKBP10*-overexpressing IMR-32 cells showed an increased number of migrated cells in the Transwell assay, further supporting an association between *FKBP10* expression and migratory capacity (Fig. 5B, Migration panels). Furthermore, Matrigel-pre-coated inserts were applied to simulate the extracellular matrix (ECM) barrier. A comparable reduction in invasive capacity was detected in *FKBP10*-deficient groups, with markedly fewer cells penetrating the Matrigel matrix (Fig. 5B). Consistent with this trend, *FKBP10* overexpression in IMR-32 cells was accompanied by enhanced penetration through the Matrigel-coated membrane, indicating increased invasive potential relative to control cells (Fig. 5B, Invasion panels). Taken together, these functional observations support an association between *FKBP10* expression and the migratory and invasive phenotypes of neuroblastoma cells *in vitro*.

To further verify the link between *FKBP10* and cell migratory and invasive phenotypes, rescue assays were conducted by ectopically overexpressing *FKBP10* in *FKBP10*-knockdown SK-N-BE(2) and SH-SY5Y cells. The impaired wound-healing capacity caused by *FKBP10* silencing was partially restored upon exogenous *FKBP10* re-expression (Fig. S4A). Likewise, Transwell migration and invasion assays revealed that re-introduction of *FKBP10* partially rescued the defective migratory and invasive phenotypes induced by *FKBP10* depletion (Fig. S4B).

**Figure 5:**
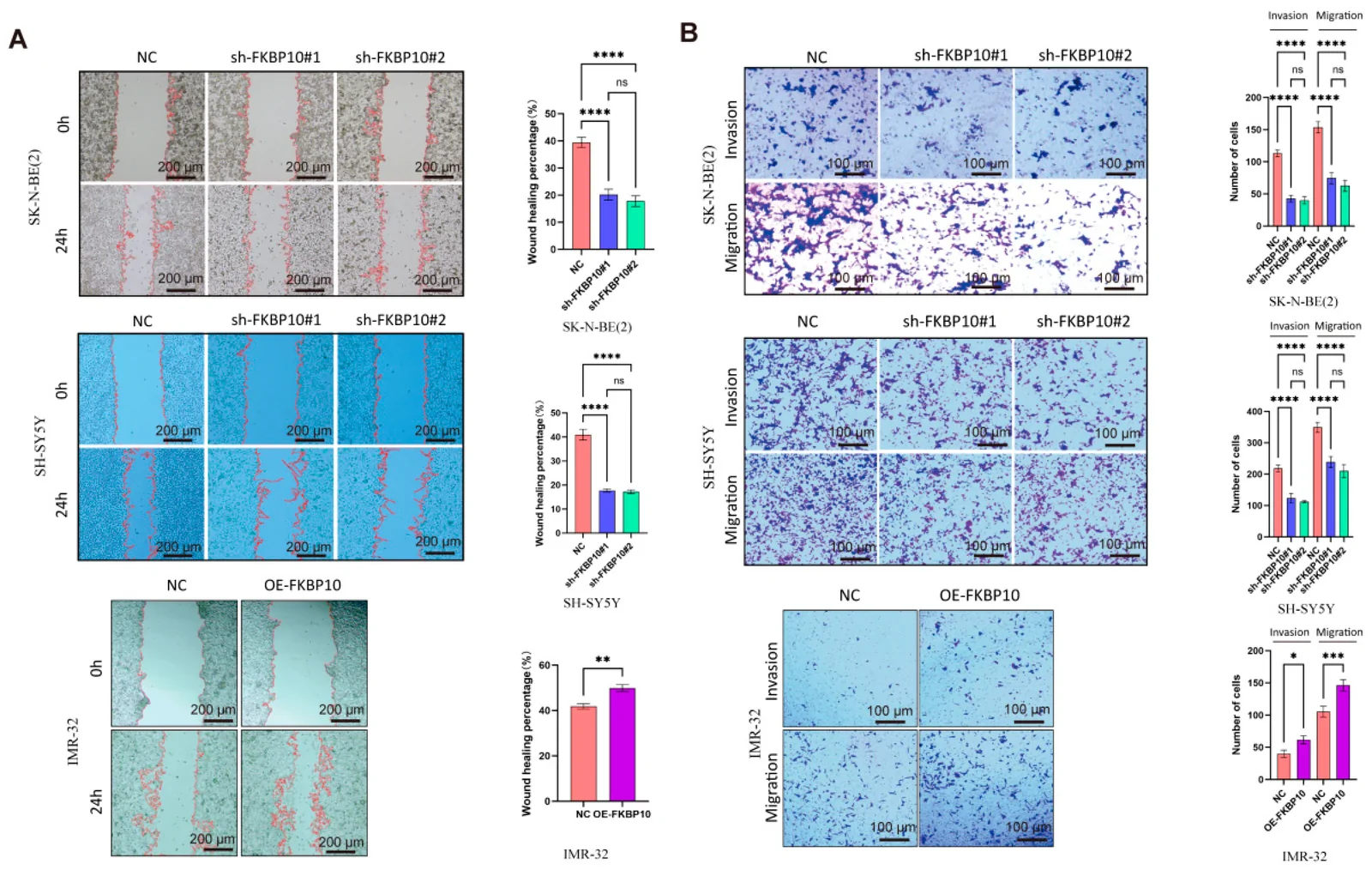
*FKBP10* promotes neuroblastoma cell migration and invasion *in vitro*. (**A**) Representative wound-healing images and quantification of wound closure in SK-N-BE(2), SH-SY5Y, and IMR-32 cells with *FKBP10* knockdown or overexpression, as indicated. Images were captured at 0 and 24 h after scratching. (**B**) Representative images and quantification of Transwell migration and invasion assays in the indicated groups. Migration assays were performed without Matrigel, whereas invasion assays were conducted using Matrigel-coated inserts. Scale bars: 100 μm. Data are presented as mean ± SD from three independent experiments. Statistical significance was determined by one-way ANOVA. *p < 0.05, **p < 0.01, ***p < 0.001, ****p < 0.0001; ns, not significant.

### 3.6 FKBP10 Is Correlated with EMT and ECM Remodeling Signatures in Neuroblastoma

Following the validation of the pro-migratory and pro-invasive phenotypes mediated by *FKBP10* in neuroblastoma cells, we further explored the potential molecular mechanisms and transcriptomic signatures underlying its oncogenic effects. Gene Set Enrichment Analysis (GSEA) was performed using cohort-based risk stratification to characterize pathway alterations linked to the high-risk phenotype. In addition, KEGG pathway enrichment analysis of genes associated with the risk signature was conducted to obtain an overview of the biological programs potentially linked to *FKBP10*-related malignant progression (Fig. S5A). This analysis revealed that the enriched pathways were mainly concentrated in tumor progression- and microenvironment-related processes, including ECM-receptor interaction, focal adhesion, the *PI3K-Akt* signaling pathway, and the *MAPK* signaling pathway, while several inflammation- and stress-associated pathways were also represented. These data suggest that the *FKBP10*-associated high-risk transcriptional context may be functionally connected with extracellular matrix organization, adhesive signaling, and pro-metastatic intracellular signaling networks. GSEA results revealed distinct pathway enrichment patterns between high- and low-risk subgroups. The high-risk subgroup exhibited significant enrichment of metastasis- and invasion-related KEGG pathways, including ECM-receptor interaction, focal adhesion, and the *TGF-β* signaling pathway, which are closely implicated in cell motility and epithelial-mesenchymal transition (EMT) modulation (Fig. S5B). In contrast, low-risk patients showed enrichment of pathways related to neuronal differentiation and physiological neuronal function, such as axon guidance and the calcium signaling pathway. These transcriptomic landscape differences indicated that the high-risk signature is tightly correlated with ECM remodeling and EMT-associated transcriptional programs.

To further clarify the clinical correlation between *FKBP10* and EMT/ECM remodeling signatures, we conducted co-expression analysis in a clinical neuroblastoma cohort (*n* = 498). Pearson correlation analysis demonstrated that *FKBP10* expression was significantly and positively correlated with canonical mesenchymal markers and core EMT-inducing transcription factors, including *CDH2*, *VIM*, *SNAI2*, and *TWIST1* (Fig. S5C). In addition, *FKBP10* levels were positively associated with multiple key ECM components and remodeling genes, including *LUM*, ACTA2, and collagen family subunits (*COL1A1*, *COL1A2*, *COL3A1*) (Fig. S5D). Collectively, these bioinformatic analyses demonstrated that elevated FKBP10 expression was closely correlated with activated EMT and ECM remodeling transcriptional profiles in neuroblastoma, providing transcriptomic clues for the pro-metastatic phenotypes observed in cell models. Based on these consistent bioinformatic correlations, we further verified these molecular alterations at the protein level using *in vitro* cellular models in the subsequent experiments.

Prompted by the suppressed migratory and invasive phenotypes following *FKBP10* depletion in neuroblastoma cells, we next examined whether *FKBP10* dysregulation was accompanied by changes in EMT- and ECM remodeling-related proteins. Since EMT and extracellular matrix remodeling are widely recognized as central processes associated with metastatic dissemination, classical epithelial/mesenchymal markers together with matrix metalloproteinases were selected for validation. Western blot analysis (Fig. 6A,B) demonstrated that *FKBP10* silencing in SK-N-BE(2) and SH-SY5Y cells correlated with elevated expression of the epithelial marker *E-Cadherin*. In parallel, the protein levels of mesenchymal markers, including *N-Cadherin* and *Vimentin*, were decreased relative to the negative control group. Consistent with the impaired invasive ability observed in functional assays, *FKBP10* knockdown also corresponded to reduced protein abundance of the critical ECM-remodeling proteases *MMP2* and *MMP9*. Collectively, these molecular alterations are highly consistent with the phenotypic changes, indicating that *FKBP10* may be implicated in modulating EMT programs and ECM proteolytic activity, thereby correlating with the metastatic behaviors of neuroblastoma cells.

**Figure 6:**
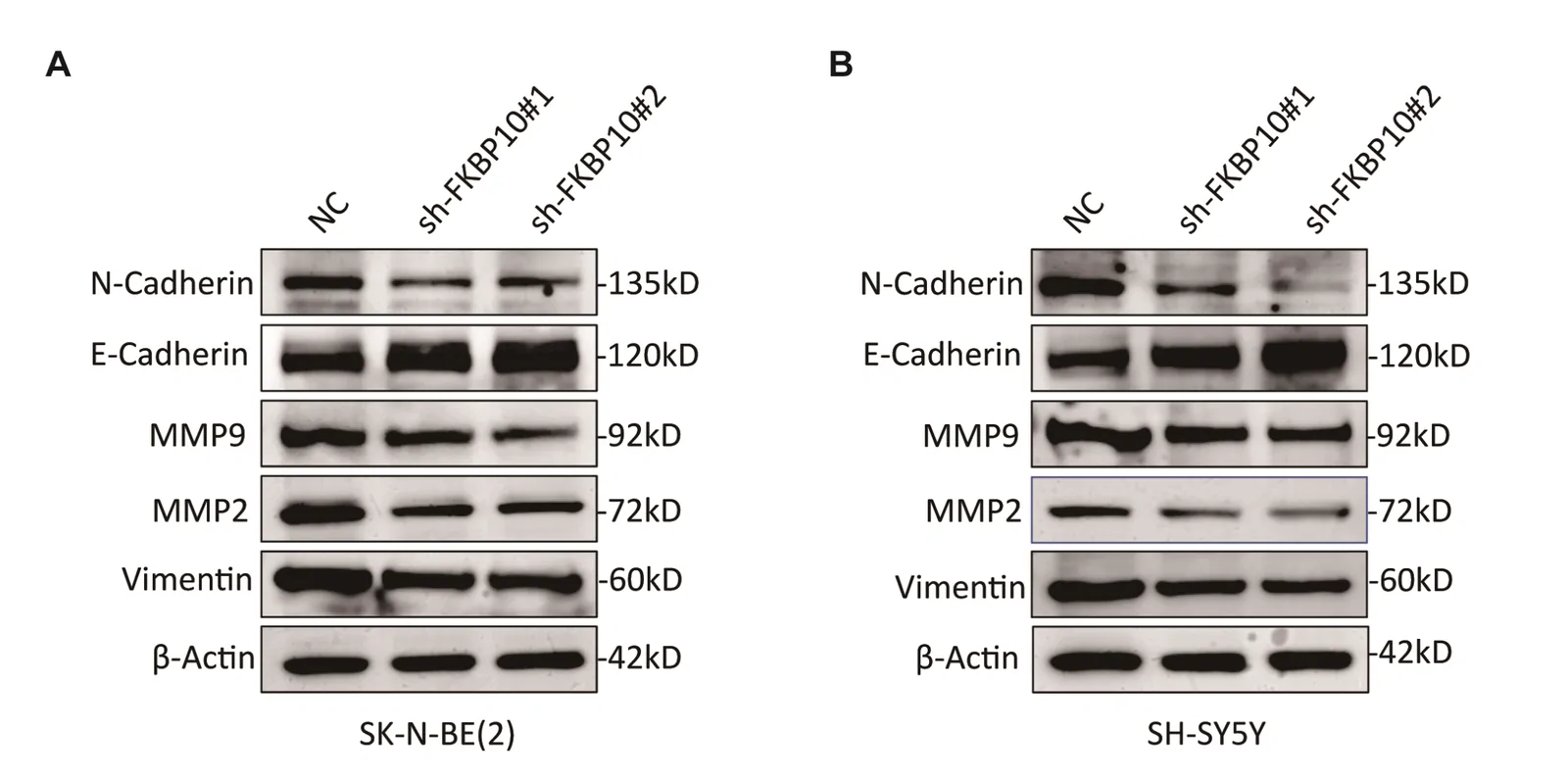
*FKBP10* knockdown modulates epithelial–mesenchymal transition (EMT) and invasion-related protein expression in neuroblastoma cells. (**A**,**B**) Western blot analysis of EMT markers (*E-cadherin*, *N-cadherin*, and *vimentin*) and matrix metalloproteinases (*MMP2* and *MMP9*) in SK-N-BE(2) (**A**) and SH-SY5Y (**B**) cells following *FKBP10* knockdown with two independent shRNAs. *β-Actin* served as the loading control.

### 3.7 FKBP10 Depletion Profoundly Impairs Neuroblastoma Tumorigenesis In Vivo

Having comprehensively elucidated the oncogenic mechanisms of *FKBP10*, specifically its role in driving proliferation and EMT, in our *in vitro* models, we next sought to determine whether these anti-tumor effects could be recapitulated in a physiologically relevant *in vivo* setting. To this end, we established a subcutaneous xenograft mouse model using the stably transduced neuroblastoma cells.

At the experimental endpoint, excised tumors from the *FKBP10*-silenced groups (*sh-FKBP10#1* and *sh-FKBP10#2*) were visibly smaller than those from the negative control group (Fig. 7A). Consistently, terminal tumor weight was significantly reduced following FKBP10 knockdown (*p* < 0.01; Fig. 7B). Longitudinal monitoring further showed that tumors with *FKBP10* depletion exhibited a slower growth rate over the 28-day observation period and reached smaller final volumes than control tumors (*p* < 0.01; Fig. 7C). These *in vivo* findings are consistent with the *in vitro* data and support an association between *FKBP10* expression and NB tumor growth.

**Figure 7:**
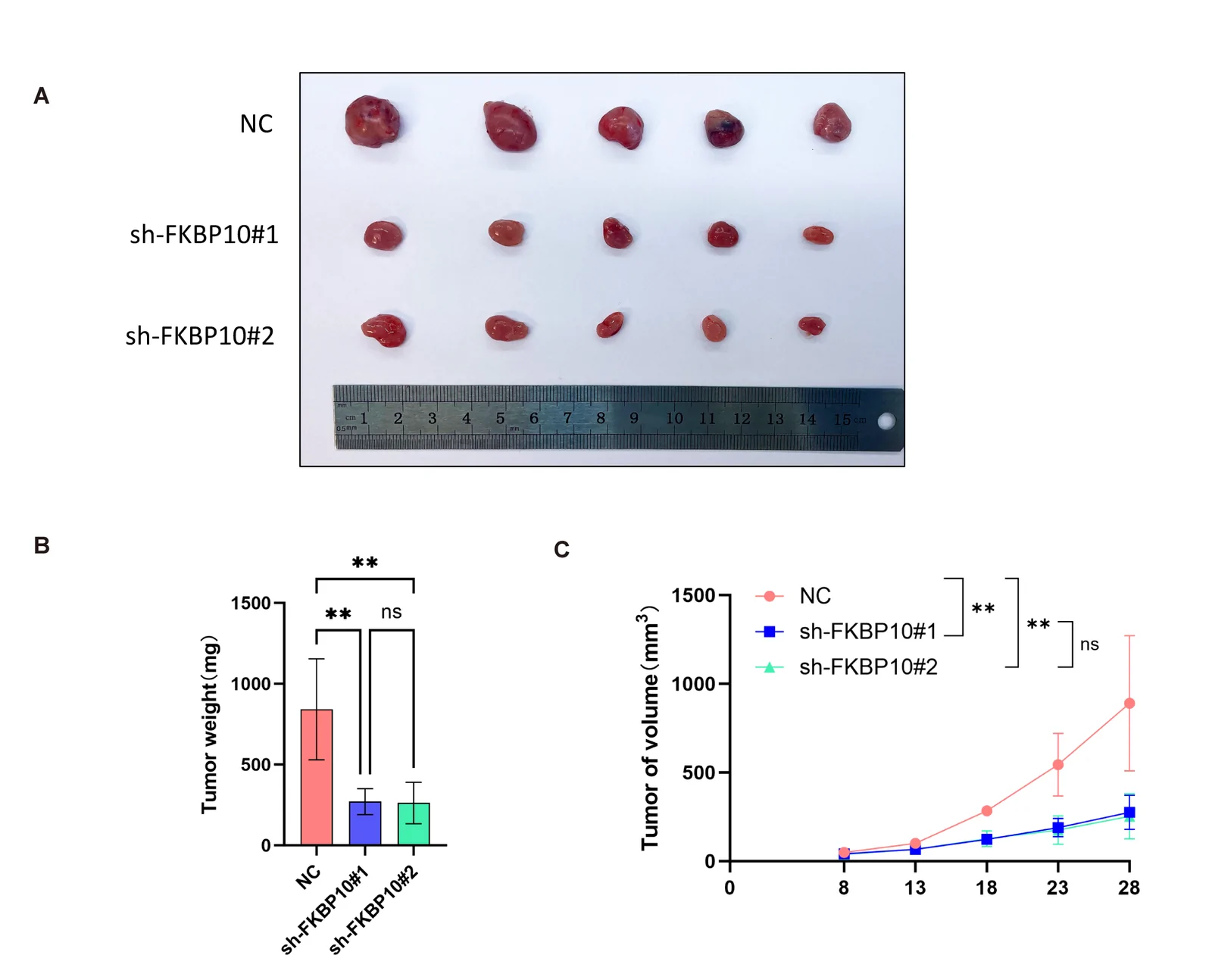
Silencing of *FKBP10* markedly inhibits neuroblastoma tumor growth in a mouse xenograft model. (**A**) Representative photographic images of excised subcutaneous xenograft tumors from nude mice injected with stable *NC*, *sh-FKBP10#1*, or *sh-FKBP10#2* cells at the experimental endpoint (day 28). (**B**) Bar graph depicting the average terminal tumor weights harvested from the respective groups. (**C**) Longitudinal tumor growth curves demonstrating the kinetic changes in tumor volume (measured in mm^3^) over the 28-day observation period. Data are presented as the mean ± SD. Statistical differences were evaluated using ANOVA. **p < 0.01; ns, not significant.

## 4 Discussion

In the present study, we systematically identified and validated *FKBP10* as a novel CAF-correlated candidate biomarker associated with malignant phenotypes in NB. Through integrated multi-cohort bioinformatic screening, weighted gene co-expression network analysis (WGCNA), machine learning-based feature selection, and *LASSO-Cox* prognostic modeling, we demonstrated that elevated *FKBP10* expression is tightly correlated with stromal activation signatures and adverse clinical prognosis in NB patients. Single-cell transcriptomic dissection further clarified that *FKBP10* exhibits a unique dual-compartment expression pattern within the NB tumor microenvironment (TME), with detectable expression in both stromal fibroblast populations and malignant NB cells. Subsequent *in vitro* functional verification, rescue phenotypic validation, mechanistic analysis, and *in vivo* xenograft animal experiments consistently illustrated that *FKBP10* dysregulation is closely associated with multiple malignant phenotypes, including NB cell proliferation, clonogenicity, migration, invasion, ECM remodeling, mesenchymal-like transition, tumor growth, and altered therapeutic sensitivity. Collectively, these multi-omics and functional lines of evidence support the notion that *FKBP10* may serve as a critical molecular linker bridging stromal microenvironment remodeling and tumor-intrinsic malignant progression in neuroblastoma.

Accumulating evidence indicates that tumor stromal activation and extracellular matrix (ECM) remodeling represent key regulatory events during tumor progression rather than merely passive secondary outcomes [23,24,25,26,36]. Excessive collagen deposition, matrix stiffening, and aberrant stromal signaling collectively establish a pro-tumor microenvironment conducive to tumor cell dissemination and adaptive survival [23,24,26,37,38]. Consistent with these established findings, our GSEA and gene co-expression analyses revealed that the high-risk NB phenotype is prominently characterized by the enrichment of metastasis-associated signaling pathways, including ECM-receptor interaction, focal adhesion, and *TGF-β* signaling. Further correlation validation confirmed that *FKBP10* expression is strongly and positively correlated with a panel of core ECM remodeling genes, including collagen family subunits (*COL1A1*, *COL1A2*, *COL3A1*), *LUM*, *DCN*, and *ACTA2*. Together with the previously reported canonical role of *FKBP10* in procollagen maturation and stromal homeostasis maintenance [30,31], our correlative transcriptomic data raise the possibility that *FKBP10* upregulation may contribute to a collagen-rich stromal niche permissive for NB progression; however, direct experimental evidence supporting this model remains to be established.

As indispensable components of the TME, CAFs can influence tumor progression through matrix remodeling, paracrine signaling, metabolic support, and therapy resistance [23,25,29]. More recent studies have further emphasized the marked heterogeneity of CAF populations and their context-dependent functions in matrix organization, immune regulation, and therapeutic response [19,20,21,22,39]. Within this framework, our data suggest that *FKBP10* may be associated with matrix-producing fibroblastic programs in NB. Importantly, our single-cell transcriptomic results revealed a critical heterogeneous expression pattern of *FKBP10* in the NB microenvironment. Different from conventional stroma-specific signature genes, *FKBP10* is detectable in both stromal fibroblasts and malignant NB cells, implying that its pro-tumor association is not merely dependent on stromal activation. Instead, *FKBP10* may operate across both stromal and tumor compartments, potentially linking extracellular matrix remodeling to tumor-intrinsic aggressiveness. Such a dual-compartment pattern may be particularly relevant in NB, where cell-state plasticity and developmental transitions strongly influence invasion, resistance, and progression [6,7,40,41]. Molecules acting in both stromal and malignant compartments may therefore serve as important mediators of tumor–microenvironment crosstalk [7,13,41]. Subsequent *in vitro* functional assays focused on malignant cells further verified the intrinsic oncogenic relevance of *FKBP10* in NB. Genetic perturbation experiments demonstrated that *FKBP10* knockdown significantly inhibits cell proliferation and colony formation, whereas *FKBP10* overexpression enhances proliferative capacity. More importantly, rescue experiments via exogenous *FKBP10* re-expression successfully reversed the impaired proliferative and clonogenic phenotypes induced by *FKBP10* silencing, confirming the cell-intrinsic oncogenic activity of *FKBP10* in NB tumor cells. It should be emphasized that current CAF-related observations are derived from transcriptome deconvolution, co-expression analysis, and single-cell annotation. Therefore, *FKBP10* should be regarded as a CAF-correlated candidate biomarker rather than a functionally validated mediator of CAF-driven tumor-stroma crosstalk.

A particularly notable finding of our study is the close association between *FKBP10* and a mesenchymal transition-like transcriptional program. *FKBP10* positively correlated with *CDH2*, *SNAI2*, *FN1*, *TWIST1*, *VIM*, and *ZEB1*. In terms of metastatic phenotypes, wound-healing and Transwell assays confirmed that *FKBP10* depletion restricts the migratory and invasive capacities of NB cells. Consistent with proliferative phenotypic rescue, ectopic *FKBP10* overexpression partially restored the defective motility caused by *FKBP10* knockdown, further supporting the functional link between *FKBP10* and NB metastatic behaviors. Mechanistically, GSEA and clinical co-expression analyses indicated that *FKBP10* is closely correlated with mesenchymal transition-related transcriptional profiles. Further Western blot validation confirmed that *FKBP10* silencing upregulates the epithelial marker *E-Cadherin*, while suppressing mesenchymal markers (*N-Cadherin*, *Vimentin*) and ECM-remodeling proteases (*MMP2*, *MMP9*). Because NB is a neural crest-derived embryonal malignancy rather than a canonical epithelial cancer, these observations are more appropriately interpreted as reflecting a mesenchymal transition-like phenotype rather than classical epithelial-mesenchymal transition. Nevertheless, the biological implication is similar: *FKBP10* appears to support a more motile, invasive, and metastasis-competent state [40,41,42,43]. This mesenchymal-like molecular reprogramming confers on tumor cells with higher motility and matrix-degrading capacity, which largely explains the pro-metastatic association of *FKBP10* in NB. The concurrent association of *FKBP10* with both ECM-remodeling genes and mesenchymal-like markers raises the possibility that *FKBP10* links intracellular phenotypic plasticity with extracellular niche remodeling during NB progression [26,40], although further mechanistic investigation is needed.

In addition to cell-intrinsic malignant phenotypes, our clinical risk model analysis revealed that the *FKBP10*-dominated high-risk signature is correlated with stromal activation, elevated TIDE scores, and predicted immune evasion, as well as reduced responsiveness to immunotherapy, chemotherapy, and targeted agents. Consistent with bioinformatic predictions, *in vitro* drug sensitivity assays further verified that *FKBP10* silencing significantly reduces the IC_50_ values of cisplatin, lapatinib, and MK-2206, indicating enhanced drug sensitivity in *FKBP10*-depleted NB cells. These translational results bridge the stromal prognostic signature with functional phenotypic changes, suggesting that *FKBP10*-related stromal remodeling and tumor-intrinsic malignant programs may collectively contribute to therapeutic resistance in high-risk NB patients. It is important to note that associations between *FKBP10* and stromal remodeling are based on correlative transcriptomic data.

*In vivo* xenograft models further validated the pro-growth association of *FKBP10* in physiological tumor microenvironments. *FKBP10* knockdown markedly suppressed subcutaneous tumor growth, accompanied by reduced tumor volume and weight throughout the observation period, which was highly consistent with our *in vitro* phenotypic observations. Collectively, our multi-level evidence demonstrates that *FKBP10* correlates with NB malignant progression by coordinating stromal ECM remodeling, mesenchymal-like phenotypic transition, tumor cell proliferation and metastasis, and therapeutic resistance.

Our findings may also have implications for the immune and stromal architecture of the NB microenvironment. Fibroblast-rich and collagen-dense stromal environments have been shown to restrict lymphocyte infiltration, reinforce suppressive myeloid programs, and contribute to resistance to immunotherapy in multiple tumor types [12,27,44,45,46]. In particular, *TGF-β*-associated stromal activation and ECM remodeling have been linked to T-cell exclusion and immune evasion [27,45,47]. Spatial and single-cell studies further suggest that discrete fibroblast states can organize local immune niches and shape the architecture of treatment-resistant microenvironments [12,20,22,48]. Given the correlation between *FKBP10* and CAF-ECM transcriptional signatures in our dataset, we hypothesize that *FKBP10*-associated stromal states may contribute to the formation of an immune-suppressive microenvironment in NB. However, our study contains no direct experimental assessment of immune cell function, spatial tissue architecture, or *in vivo* immunotherapy responses. Interpretations concerning immune exclusion remain hypothesis-generating and require dedicated validation using spatial transcriptomics, multiplexed tissue imaging, or immunocompetent preclinical models.

From a translational perspective, *FKBP10* represents an interesting candidate because it lies at the intersection of stromal remodeling and malignant progression. Broad CAF depletion has produced mixed and sometimes paradoxical effects in several cancers, likely reflecting the functional heterogeneity of fibroblast populations [15,21,49]. In this context, selective targeting of specific stromal-associated effectors may be more feasible than indiscriminate depletion of all CAF populations [17,18,39]. *FKBP10* is of particular interest because prior studies have implicated it in collagen processing, fibrotic remodeling, immune-related stromal phenotypes, and tumor-promoting activities in non-NB settings [31,32,33,34,35,50]. Our findings extend these associations to NB and support further investigation of *FKBP10* as a candidate biomarker and potential therapeutic target.

This study has several limitations that should be acknowledged. First, although multi-cohort validation, single-cell profiling and phenotypic rescue experiments support the tumor-promoting properties of *FKBP10*, most transcriptomic analyses remain correlative; direct causal regulatory mechanisms require additional molecular experimental validation. Second, the present work mainly characterizes cell-intrinsic functions of *FKBP10* in malignant NB cells; the biological functions of *FKBP10* within CAFs and stroma-immune crosstalk remain unaddressed. Third, conclusions regarding immune evasion and immunotherapy resistance are derived solely from computational prediction and lack validation from spatial immune profiling or independent immunotherapy cohorts. Finally, the subcutaneous xenograft model cannot fully recapitulate the metastatic niche and immunocompetent background of clinical neuroblastoma. Future studies incorporating spatial transcriptomics, stroma-tumor co-culture systems, and metastatic animal models are needed to dissect the dual-compartment functions of *FKBP10* in tumor cells and stromal compartments.

## 5 Conclusion

Our study identifies *FKBP10* as a clinically relevant stroma-associated candidate factor linked to aggressive neuroblastoma. *FKBP10* expression is correlated with CAF enrichment signatures, ECM remodeling, mesenchymal-like reprogramming, and bioinformatically predicted immune dysfunction and therapeutic resistance. Functional assays demonstrated that *FKBP10* promotes NB cell proliferation, migration, and invasion *in vitro* and tumor growth *in vivo*. These findings support *FKBP10* as a candidate prognostic biomarker and a potential therapeutic target for high-risk NB. Further mechanistic studies of tumor-stroma interactions are warranted to clarify its precise roles, particularly its functional contribution within stromal compartments.

## Data Availability

The data that support the findings of this study are available from the Corresponding authors upon reasonable request.

## Supplementary Materials

The supplementary material is available online at https://www.techscience.com/doi/10.32604/or.2026.087891/s1.
