# Supplementary material for: Integrative Multi-Omics and Single-Cell Analyses Identify FKBP10 as a CAF-Associated Biomarker Linked to ECM Remodeling and Aggressiveness in Neuroblastoma
Source: Oncol Res. 2026 Oct 28;34(11):23. doi: 10.32604/or.2026.087891 (PMC13732391; doi:10.32604/or.2026.087891)
Supplement: Supplementary file 1 [file OncolRes-34-87891-s001.zip › TSP_OR_87891-s001.docx]

**Supplementary Materials**

**Figure S1.** Identification of stromal/cancer-associated fibroblast (CAF)-related co-expression modules in neuroblastoma by WGCNA. (A) For the GSE45547 cohort, the left and middle panels show the scale-free topology fit index and mean connectivity across different soft-thresholding powers, and the right panel shows the module–trait relationship heatmap. (B) For the GSE49710 cohort, the left and middle panels show the scale-free topology fit index and mean connectivity across different soft-thresholding powers, and the right panel shows the module–trait relationship heatmap. (C) For the E-MTAB cohort, the left and middle panels show the scale-free topology fit index and mean connectivity across different soft-thresholding powers, and the right panel shows the module–trait relationship heatmap. In each heatmap, rows represent module eigengenes, and columns represent the stromal-related traits estimated by EPIC, MCPcounter, xCell, and StromalScore. Values in each cell indicate the Pearson's correlation coefficient and the corresponding *P* value; red denotes positive correlation, and blue denotes negative correlation.

**Figure S2.** *FKBP10* re-expression partially rescues the impaired proliferative capacity induced by *FKBP10* knockdown in neuroblastoma cells. (A) Western blot analysis confirming *FKBP10* knockdown and re-expression in SK-N-BE(2) and SH-SY5Y cells; β-Actin served as the loading control. (B) Representative colony formation images and corresponding quantification in the indicated groups. (C) Representative EdU staining images and quantification of EdU-positive cells in SK-N-BE(2) and SH-SY5Y cells under the indicated treatments. Data are presented as mean ± SD. Statistical significance was assessed by one-way ANOVA. ***P* < 0.01, ****P* < 0.001, *****P* < 0.0001.

**Figure S3.** *FKBP10* knockdown enhances drug sensitivity in SH-SY5Y cells. (A) Dose–response curve for MK-2206 in SH-SY5Y-NC cells. (B) Dose–response curve for MK-2206 in SH-SY5Y-sh-*FKBP10#1* cells. (C) Dose–response curve for lapatinib in SH-SY5Y-NC cells. (D) Dose–response curve for lapatinib in SH-SY5Y-sh-*FKBP10*#1 cells. (E) Dose–response curve for cisplatin in SH-SY5Y-NC cells. (F) Dose–response curve for cisplatin in SH-SY5Y-sh-*FKBP10*#1 cells. Dose–response curves for cisplatin in SH-SY5Y-NC and SH-SY5Y-sh-*FKBP10#1* cells, respectively. Cell viability was assessed following treatment with the indicated concentrations, and IC_50_ values are shown in each panel. Depletion of *FKBP10* reduced the half-maximal inhibitory concentration (IC50) values of all three agents, indicating increased drug sensitivity.

**Figure S4.** *FKBP10* restoration partially rescues the suppressed migratory and invasive phenotypes induced by *FKBP10* knockdown in neuroblastoma cells. (A) Representative wound-healing images and quantification of wound closure in SK-N-BE(2) and SH-SY5Y cells under the indicated treatments. (B) Representative Transwell migration and invasion images and corresponding quantification in SK-N-BE(2) and SH-SY5Y cells. Scale bar, 100 μm. Data are presented as mean ± SD. **P*<0.05, ***P*<0.01, ****P*<0.001, *****P* < 0.0001.

**Figure S5.** Functional enrichment and correlation analyses associated with *FKBP10* expression. (A) Bubble plot showing KEGG enrichment analysis of *FKBP10*-associated genes. Dot size represents gene count and color indicates adjusted *P* value. (B) GSEA showing representative pathways significantly enriched in the high-risk and low-risk groups. (C) Co-expression analyses of *FKBP10* with EMT-related genes in the GSE49710 neuroblastoma cohort. (D) Co-expression analyses of *FKBP10* with bulk RNA-based stromal and ECM-related signatures in the GSE49710 neuroblastoma cohort, including COL1A1, COL1A2, COL3A1, DCN, FAP, BGN, LUM, ACTA2, and ECM remodeling score.
